# Tissue-Wide Effects Override Cell-Intrinsic Gene Function in Radial Neuron Migration

**DOI:** 10.1093/oons/kvac009

**Published:** 2022-07-07

**Authors:** Andi H Hansen, Florian M Pauler, Michael Riedl, Carmen Streicher, Anna Heger, Susanne Laukoter, Christoph Sommer, Armel Nicolas, Björn Hof, Li Huei Tsai, Thomas Rülicke, Simon Hippenmeyer

**Affiliations:** Institute of Science and Technology Austria, Am Campus 1, 3400 Klosterneuburg, Austria; Institute of Science and Technology Austria, Am Campus 1, 3400 Klosterneuburg, Austria; Institute of Science and Technology Austria, Am Campus 1, 3400 Klosterneuburg, Austria; Institute of Science and Technology Austria, Am Campus 1, 3400 Klosterneuburg, Austria; Institute of Science and Technology Austria, Am Campus 1, 3400 Klosterneuburg, Austria; Institute of Science and Technology Austria, Am Campus 1, 3400 Klosterneuburg, Austria; Institute of Science and Technology Austria, Am Campus 1, 3400 Klosterneuburg, Austria; Institute of Science and Technology Austria, Am Campus 1, 3400 Klosterneuburg, Austria; Institute of Science and Technology Austria, Am Campus 1, 3400 Klosterneuburg, Austria; Picower Institute for Learning and Memory, MIT, Cambridge, MA 02139, USA; Department of Biomedical Sciences, University of Veterinary Medicine Vienna, 1210 Vienna, Austria; Institute of Science and Technology Austria, Am Campus 1, 3400 Klosterneuburg, Austria

**Keywords:** cell-autonomous gene function, 4D live-imaging, single-cell genetics, neuronal migration, mosaic analysis with double markers (MADM), cerebral cortex development, non-cell-autonomous effects

## Abstract

The mammalian neocortex is composed of diverse neuronal and glial cell classes that broadly arrange in six distinct laminae. Cortical layers emerge during development and defects in the developmental programs that orchestrate cortical lamination are associated with neurodevelopmental diseases. The developmental principle of cortical layer formation depends on concerted radial projection neuron migration, from their birthplace to their final target position. Radial migration occurs in defined sequential steps, regulated by a large array of signaling pathways. However, based on genetic loss-of-function experiments, most studies have thus far focused on the role of cell-autonomous gene function. Yet, cortical neuron migration *in situ* is a complex process and migrating neurons traverse along diverse cellular compartments and environments. The role of tissue-wide properties and genetic state in radial neuron migration is however not clear. Here we utilized mosaic analysis with double markers (MADM) technology to either sparsely or globally delete gene function, followed by quantitative single-cell phenotyping. The MADM-based gene ablation paradigms in combination with computational modeling demonstrated that global tissue-wide effects predominate cell-autonomous gene function albeit in a gene-specific manner. Our results thus suggest that the genetic landscape in a tissue critically affects the overall migration phenotype of individual cortical projection neurons. In a broader context, our findings imply that global tissue-wide effects represent an essential component of the underlying etiology associated with focal malformations of cortical development in particular, and neurological diseases in general.

## Teaser

The assembly of the mammalian brain is a complex process and depends on tightly regulated developmental and genetic programs. The coordinated process of nerve cell (neuron) migration is essential to home neurons into their correct position, which is vital for normal brain development. Disturbance of the neuron migration process at any point of development leads to severe brain malformations resulting in devastating disease. To date, studies have mainly focused on cell-intrinsic gene functions controlling neuronal migration. Therefore, we got very little knowledge about the possible contribution of the cellular surrounding and tissue-wide effects. The scale and nature of such global tissue-wide effects remain completely unclear. We thus established genetic platforms based on mosaic analysis with double markers to visualize and quantify tissue-wide effects in a defined genetic context and at single cell resolution. We found a critical predominant role of the genetic landscape and state of cellular environment affecting overall neuron migration properties. In a broader context, our results suggest that global tissue-wide effects are a major component of the underlying etiology of neurological diseases such as focal malformations of cortical development.

## INTRODUCTION

The mammalian neocortex, arranged in a six-layered structure, executes essential higher order cognitive brain functions. The laminated organization emerges during embryonic development and instructs the wiring diagram of cortical microcircuits [26, 48]. Deficits in the developmental programs orchestrating cortical layering represent a key underlying mechanism of neurodevelopmental disorders including cortical malformation [22, 30, 38].

A cardinal feature of cortical layering during development is the temporally sequential arrangement of the distinct layers in an ‘inside-out’ fashion [2, 52, 80]. As such, progressively later born, radial glial cell (RGC)-derived, projection neurons migrate out radially past earlier born cohorts to gradually build up the inversely arranged laminae. Seminal live-imaging experiments at single-cell resolution revealed that radial migration of cortical projection neurons occurs in a defined sequence [54, 57, 58, 79]. First, newly-born projection neurons acquire a bipolar morphology and migrate from the ventricular zone (VZ) to the subventricular zone (SVZ) where they adopt a multipolar shape [76]. While in the SVZ, multipolar neurons move slowly tangentially, a process that may be required to explore the extracellular environment for putative polarity-inducing cues [14, 37]. Next, multipolar neurons switch back to a bipolar state with the ventricle-oriented process developing into the axon. Bipolar projection neurons reattach to the radial glial fiber, migrate through the intermediate zone (IZ) and enter the cortical plate (CP) by using a locomotion mode [29, 41]. Once the locomoting neurons reach the most superficial layer, they detach from the radial glial fiber and perform terminal somal translocation to settle in their target position [18, 71, 72].

In the past decades, a large collection of signaling pathways including extracellular cues/receptors, cell adhesion molecules and their receptors, endocytic/exocytic regulators, intracellular signal transduction machinery and transcription factors have been implicated in controlling the discrete sequential steps of radial projection neuron migration in healthy and disease conditions [4, 9, 16, 27, 30, 31, 42, 64]. Thus far, most studies using loss-of-function paradigms focused almost exclusively on the cell-autonomous role of the genes encoding the above regulatory cues. Yet, accumulating evidence suggests that non-cell-autonomous tissue-wide properties could substantially impact and/or contribute to the regulation of radial projection neuron migration [6, 18, 19, 21 24, 25, 28, 31, 32, 55, 68, 82–84]. The nature and dynamics of such global tissue-wide effects remain however unclear.

In our study, we addressed this issue in a quantitative manner at single-cell level and set out to establish experimental genetic paradigms enabling the separation of intrinsic cell-autonomous gene function from the contribution of tissue-wide effects. To this end we utilized mosaic analysis with double markers (MADM) technology [12, 86], which provides a quantitative platform *in situ* to (i) monitor neuronal migration with exquisite single-cell resolution and (ii) probe the role of the genetic landscape and cellular environment on individual migrating neurons in a defined genetic context. Based on results from holistic single-cell phenotypic analysis upon loss of gene function, we provide evidence that global tissue-wide effects predominate the overall cell-autonomous phenotype of migrating projection neurons.

## RESULTS

### MADM-based platform to probe cell-autonomous candidate gene function and the contribution of global tissue-wide effects at single-cell level

**Figure 1:**
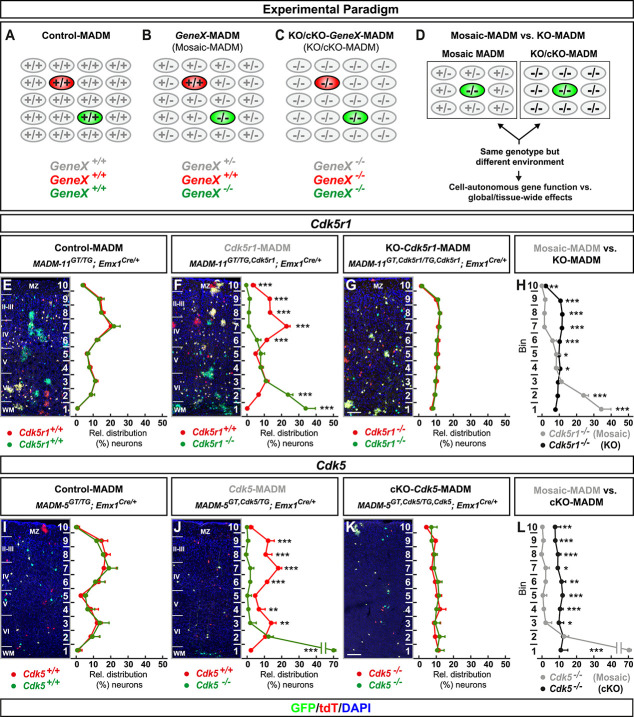
MADM analysis reveals that global tissue-wide effects predominates the cell-autonomous phenotype due to loss of p35/CDK5. (**A**–**D**) Experimental MADM paradigm to genetically dissect cell-autonomous gene function and non-cell-autonomous effects. (A) Control (control-MADM: all cells *GeneX*^+/+^); (B) sparse genetic mosaic (*GeneX*-MADM: only green cells are *GeneX*^−/−^ mutant, red cells are *GeneX*^+/+^ in an otherwise heterozygous *GeneX*^+/−^ environment); (C) global/whole-tissue gene knockout (KO/cKO-*GeneX*-MADM: all cells mutant); (D) direct phenotypic comparison of mutant cells in *GeneX*-MADM (mosaic MADM) to mutant cells in KO/cKO-*gene*-MADM (KO/cKO-MADM). Any significant difference in their respective phenotypes implies non-cell-autonomous effects. (**E–H**) Analysis of green (GFP^+^) and red (tdT^+^) MADM-labeled projection neurons in (E) control-MADM (*MADM-11^GT/TG^;Emx1^Cre/+^*); (F) *Cdk5r1*-MADM (*MADM-11^GT/TG,Cdk5r1^;Emx1^Cre/+^*); and (G) KO-*Cdk5r1*-MADM (*MADM-11^GT,Cdk5r1/TG,Cdk5r1^;Emx1^Cre/+^*). Relative distribution (%) of MADM-labeled projection neurons is plotted in ten equal zones across the cortical wall. (H) Direct distribution comparison of *Cdk5r1^−/−^* mutant cells in *Cdk5r1*-MADM (grey) versus KO-*Cdk5r1*-MADM (black) distribution. (**I–L**) Analysis of green (GFP^+^) and red (tdT^+^) MADM-labeled projection neurons in (I) control-MADM (*MADM-5^GT/TG^;Emx1^Cre/+^*); (J) *Cdk5*-MADM (*MADM-5^GT/TG,Cdk5^;Emx1^Cre/+^*); and (K) KO-*Cdk5*-MADM (*MADM-11^GT,Cdk5/TG,Cdk5^;Emx1^Cre/+^*). Relative distribution (%) of MADM-labeled projection neurons is plotted in 10 equal zones (1–10) across the cortical wall. (L) Direct distribution comparison of *Cdk5^−/−^* mutant cells in *Cdk5*-MADM (grey) versus KO-*Cdk5*-MADM (black) distribution. Nuclei were stained using DAPI (blue). N = 3 for each genotype with 10 (MADM-11) and 20 (MADM-5) hemispheres analysed. Data indicate mean ± SD, ^*^P < 0.05, ^**^P < 0.01 and ^***^P < 0.001. Scale bar: 100 μm. Marginal zone (MZ), cortical layers (II–VI), white matter (WM).

In order to establish a quantitative assay to dissect cell-autonomous gene function and to measure the contribution of global tissue-wide cues/properties with single-cell resolution, we conceived a platform consisting of three genetic MADM-based paradigms: (i) control-MADM; (ii) *GeneX*-MADM (mosaic-MADM); and (iii) KO/cKO-*GeneX*-MADM (KO/cKO-MADM) (Fig. 1A–D and Fig. S1). In control-MADM, mice carrying MADM cassettes on a particular chromosome were combined with *Emx1*-Cre driver to generate experimental MADM mice with sparse fluorescent labeling in cortical projection neurons. Since these control-MADM mice did not carry any loss-of-function (LOF) allele all cells were considered as ‘control’ with some sparse cells expressing the fluorescent GFP and tdT markers enabling single-cell tracing. In *GeneX*-MADM with *Emx1*-Cre, a genetic mutation was coupled to one MADM cassette by meiotic recombination (see Materials and Methods [1, 12] for details) in a way that sparse homozygous mutant cells were always labeled in green (GFP^+^) and wild-type cells in red (tdT^+^), respectively. In KO/cKO-*GeneX*-MADM with *Emx1*-Cre, the mutant allele of the gene of interest was coupled to both MADM cassettes resulting in full (KO) or conditional (cKO) tissue-specific knockout (*Emx1^+^* projection neuron lineage) with sparse fluorescent labeling in respective experimental MADM mice. The key rationale in our overall assay was based on the following assumptions. First, in sparse genetic mosaic-MADM (where we ablated candidate gene function in just very few GFP^+^ migrating neurons), the observed phenotype of homozygous mutant cells implied the cell-autonomous gene function when compared to tdT^+^ control cells. Second, the phenotype of individual mutant GFP^+^ cells in an all mutant environment (KO/cKO-MADM), of the same candidate gene as above, reflected the combination of (i) cell-autonomous loss of gene function; plus (ii) global community effects in response to the loss of gene function in the vast majority of (if not all) cortical projection neurons across the entire tissue. Third, any quantitatively significant difference in the observed single-cell mutant phenotype in mosaic-MADM when compared to KO/cKO-MADM indicated non-cell-autonomous and thus global tissue-wide effects (Fig. 1D).

### Global tissue-wide effects predominates the cell-autonomous phenotype due to loss of p35/CDK5

The role of the p35/CDK5 signaling pathway in cortical projection neuron migration has been studied extensively [13, 23, 25, 29, 40, 59]. Thus with ample information available, based on LOF studies, this pathway provided an ideal case to dissect cell-autonomous requirement and the contribution of tissue-wide effects due to LOF. We first analysed the single-cell phenotype upon ablation of p35 (encoded by *Cdk5r1*, located on chr.11), the main activator of CDK5. (Fig. S2A). Therefore, we generated control-MADM (*MADM-11^GT/TG^;Emx1^Cre/+^*), *Cdk5r1*-MADM (*MADM-11^GT/TG,Cdk5r1^;Emx1^Cre/+^*) and KO-*Cdk5r1*-MADM (*MADM-11^GT,Cdk5r1/TG,Cdk5r1^;Emx1^Cre/+^*). Next we quantified the relative distribution of red and green MADM-labeled cortical projection neurons (terminal position) in somatosensory cortex at postnatal day (P) 21 to assess overall migration capacity in the above MADM paradigms (Fig. 1E–G). In control-MADM red and green (both control) projection neurons distributed similarly but in a defined fashion (relatively higher number in upper layers) across 10 zones as previously reported [32] (Fig. 1E). In *Cdk5r1*-MADM, the green homozygous *Cdk5r1*^−/−^ mutant neurons accumulated in lower layers, indicative of the cell-autonomous requirement for radial migration [23, 25], while red control neurons showed a distribution pattern similar to red/green cells in control-MADM (Fig. 1F). In contrast, red and green (both homozygous *Cdk5r1*^−/−^ mutant) neurons in KO-*Cdk5r1*-MADM showed similar pattern when compared to each other but clearly different distribution pattern when compared to control and mutant cells in control-MADM and/or *Cdk5r1*-MADM, respectively (Fig. 1G). Strikingly, the distribution of green homozygous *Cdk5r1*^−/−^ mutant neurons in *Cdk5r1*-MADM was significantly distinct when compared to green homozygous *Cdk5r1*^−/−^ mutant neurons in KO-*Cdk5r1*-MADM despite that both cell populations had the same genotype (Fig. 1H). Thus, the cell-autonomous phenotype (i.e. terminal location), of *Cdk5r1*^−/−^ mutant cortical projection neurons, was significantly influenced by the genetic tissue-wide landscape.

To identify the layer specificity of the MADM-labelled neurons in all three genotypes we applied immunohistochemistry for the markers Cux1, FoxP2 and Ctip2 (Fig. S3). While mutant neurons in the *Cdk5r1*-MADM were mainly distributed in the lower zones of the cortical wall (Fig. S3B), we observed a dispersion of upper layer neurons in the KO-*Cdk5r1*-MADM (Fig. S3C). Moreover, the lower layer markers revealed an inverted distribution with a dispersion of lower layer neurons in the upper half of the cortical wall in the KO-*Cdk5r1*-MADM, when compared to control-MADM (Fig. S3E–L). Nevertheless, in the *Cdk5r1*-MADM, a portion of FoxP2 and Ctip2-positive mutant neurons were ectopically located in the lowest zones (Fig. S3F–S3J).

To corroborate the above finding we established a second MADM platform by using MADM cassettes inserted in chr.5 (i.e. MADM-5) to more directly assay the consequences of *Cdk5* (located on chr.5) loss of function (Fig. S2B). We thus generated control-MADM (*MADM-5^GT/TG^;Emx1^Cre/+^*), *Cdk5*-MADM (*MADM-5^GT,Cdk5/TG^;Emx1^Cre/+^*) and cKO-*Cdk5*-MADM (*MADM-5^GT,Cdk5/TG,Cdk5^;Emx1^Cre/+^*) and analysed the terminal distribution of red and green cells across the somatosensory cortex at P15 (since cKO-*Cdk5*-MADM animals tend to die soon thereafter). Again, in control-MADM, red and green cells (both wild-type) distributed similarly when compared to each other, and similarly when compared to red and green cells in control-MADM using MADM-11 (Fig. 1E and I). In mosaic *Cdk5*-MADM, green mutant *Cdk5*^−/−^ cells accumulated in lower layers and the white matter (Fig. 1J). The migration phenotype of *Cdk5*^−/−^ cells in *Cdk5*-MADM appeared slightly stronger than the one of *Cdk5r1*^−/−^ cells in *Cdk5r1*-MADM (Fig. 1F and J). The small phenotypic difference could presumably be due to possible compensation by p39 upon loss of p35 and thus some minor residual CDK5 activity in *Cdk5r1*^−/−^ cells [40, 75]. In any case, red and green (both homozygous *Cdk5*^−/−^ mutant) neurons in KO-*Cdk5*-MADM showed clearly different distribution patterns when compared to control and mutant cells in control-MADM and/or *Cdk5*-MADM, respectively (Fig. 1I, J and K). Again, the distribution of green, homozygous *Cdk5*^−/−^ mutant neurons, in *Cdk5*-MADM, was significantly distinct when compared to green homozygous *Cdk5*^−/−^ mutant neurons in KO-*Cdk5*-MADM despite that both cell populations had the same genotype (Fig. 1L). Altogether, our data indicate that the state of the genetic environment contributes critically to the overall phenotype of individual cells.

### Developmental progression of tissue-wide effects impacting phenotypic manifestation upon sparse and global KO of p35/CDK5

**Figure 2:**
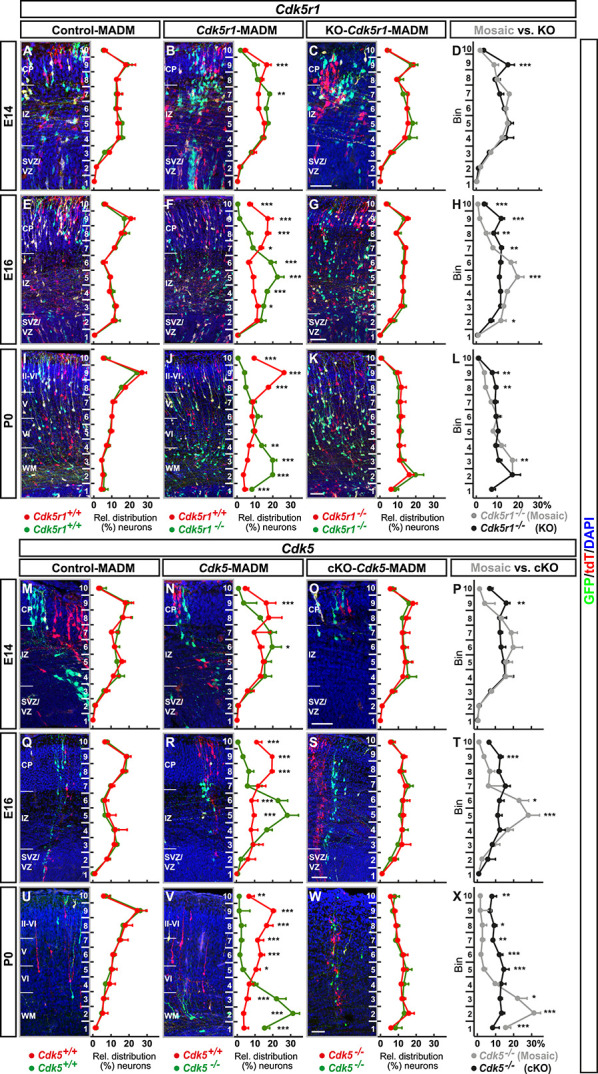
Developmental time course analysis of MADM-based sparse and global KO of p35/CDK5. (**A–L**) Analysis of green (GFP^+^) and red (tdT^+^) MADM-labeled projection neurons in (A, E, I) control-MADM (*MADM-11^GT/TG^;Emx1^Cre/+^*); (B, F, J) *Cdk5r1*-MADM (*MADM-11^GT/TG,Cdk5r1^;Emx1^Cre/+^*); and (C, G, K) KO-*Cdk5r1*-MADM (*MADM-11^GT,Cdk5r1/TG,Cdk5r1^; Emx1^Cre/+^*) at E14 (A–D), E16 (E–H) and P0 (I–L). Relative distribution (%) of MADM-labeled projection neurons is plotted in ten equal zones across the developing cortical wall. (D, H, L) Direct distribution comparison of *Cdk5r1^−/−^* mutant cells at E14 (D), E16 (H) and P0 (L) in *Cdk5r1*-MADM (grey) versus KO-*Cdk5r1*-MADM (black) distribution. (**M–X**) Analysis of green (GFP^+^) and red (tdT^+^) MADM-labeled projection neurons in (M, Q, U) control-MADM (*MADM-11^GT/TG^;Emx1^Cre/+^*); (N, R, V) *Cdk5*-MADM (*MADM-11^GT/TG,Cdk5^;Emx1^Cre/+^*); and (O, S, W) KO-*Cdk5*-MADM (*MADM-11^GT,Cdk5/TG,Cdk5^; Emx1^Cre/+^*) at E14 (M–P), E16 (Q–T) and P0 (U–X). Relative distribution (%) of MADM-labeled projection neurons is plotted in 10 equal zones across the developing cortical wall. (P, T, X) Direct distribution comparison of *Cdk5^−/−^* mutant cells at E14 (P), E16 (T), and P0 (X) in *Cdk5*-MADM (grey) versus KO-*Cdk5*-MADM (black) distribution. Nuclei were stained using DAPI (blue). N = 3 for each genotype with 10 (MADM-11) and 20 (MADM-5) hemispheres analysed. Data indicate mean ± SD, ^*^P < 0.05, ^**^P < 0.01 and ^***^P < 0.001. Cortical plate (CP), intermediate zone (IZ), subventricular zone/ventricular zone (SVZ/VZ), cortical layers (II–VI), white matter (WM). Scale bars: 100 μm.

To determine the emergence of non-cell-autonomous tissue-wide effects influencing radial neuron migration we pursued developmental time course analysis. We utilized the same MADM-based paradigms as described in the above section to visualize the single-cell phenotype upon sparse and global elimination of *Cdk5r1* (Fig. 2A–L) and *Cdk5* (Fig. 2M–X), respectively. At embryonic day (E) 14 no phenotypic difference—i.e. relative vertical distribution of mutant cells in mosaic versus cKO/KO—in VZ/SVZ and IZ was observed. However, small but significant differences were apparent in the upper zones, corresponding to the emerging CP, in both *Cdk5r1* and *Cdk5* comparative sparse and global MADM deletion paradigms (Fig. 2D and P). At E16 (Figs 2E–F and 2Q–R) and P0 (Figs 2I–J and 2U–V) time points, based on sparse mosaic MADM paradigms, the cell-autonomous phenotypes demonstrated critical requirement for *Cdk5r1/Cdk5* in migrating projection neurons to enter the developing cortical plate. In effect, although mutant cells could migrate from the VZ/SVZ through IZ, *Cdk5r1^−/−^* and *Cdk5^−/−^* mutant cells accumulated below the CP. The phenotypic manifestation became progressively stronger as the development of the CP proceeded. In contrast, mutant cells upon global *Cdk5r1/Cdk5* ablation in KO/cKO appeared to distribute more evenly across the developing cortical wall (Fig. 2G, K, S and W). Thus, the emerging cell-autonomous phenotype upon *Cdk5r1/Cdk5* ablation (deficit to enter the developing CP) was significantly affected by the global genetic constitution of the developing cortical wall.

### Distinct projection neuron migration dynamics upon sparse and global KO of p35/CDK5

**Figure 3:**
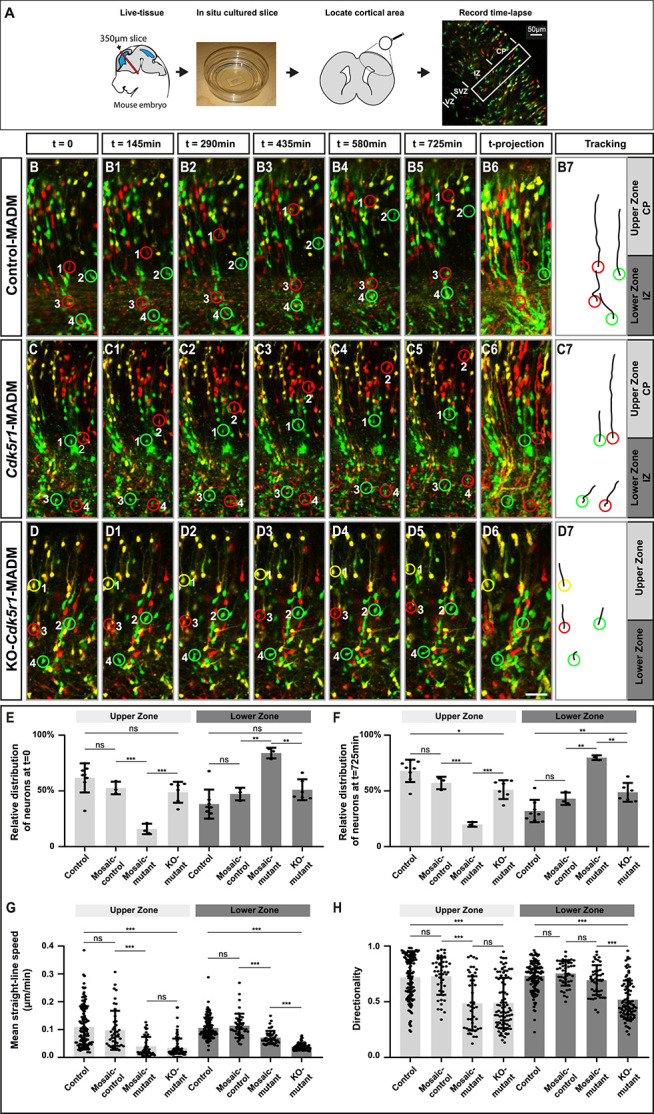
Projection neuron migration dynamics upon sparse and global KO of p35/CDK5. (**A**) Experimental setup for time-lapse imaging of MADM-labeled neurons in developing somatosensory cortex at E16. (**B–D**) Time-lapse imaging of (B-B5) control-MADM (*MADM-11^GT/TG^;Emx1^Cre/+^*), (C-C5) *Cdk5r1*-MADM (*MADM-11^GT/TG,Cdk5r1^;Emx1^Cre/+^*) and (D-D5) KO-*Cdk5r1*-MADM (*MADM-11^GT,Cdk5r1/TG,Cdk5r1^; Emx1^Cre/+^*). (B6, C6, D6) 12 h time projection of sequential images in IZ (lower zone) and emerging CP (upper zone) at 15 min frame rate. (B7, C7, D7) Tracking trajectories of indicated neurons (red and green rings) in control-MADM (B7), *Cdk5r1*-MADM (C7) and KO-*Cdk5r1*-MADM (D7). (**E**) Relative distribution of neurons at the start of the time-lapse t = 0 for each replicate time-lapse per genotype. (**F**) Relative distribution of neurons at the end of the time-lapse t = 725 min for each replicate time-lapse per genotype. (**G**) Mean straight-line speed of the top 15 tracks per replicate time-lapse per genotype. (**H**) Directionality of the top 15 tracks per replicate time-lapse per genotype. N = 3 videos from >2 independent animals. Data indicate mean ± SD, ^*^P < 0.05, ^**^P < 0.01 and ^***^P < 0.001. Scale bar: 40 μm.

To directly assess cortical projection neuron migration dynamics we measured physical movement *in-situ* by time-lapse imaging (Fig. 3A). We analysed neuronal migration in embryonic brain slices at E16 when cortical projection neurons were located at all stages in their sequential radial migration trajectory. We capitalized upon the exquisite single-cell resolution in the three—control-MADM (Fig. 3B, Movie S1), *Cdk5r1*-MADM (Fig. 3C, Movie S2) and KO-*Cdk5r1*-MADM (Fig. 3D, Movie S3)—experimental paradigms and recorded confocal images at 15 min intervals over an extended period of >15 h. Frames were generated from individual confocal stacks and processed for analysis (see Materials and Methods for details). From all imaging data, we could systematically analyse migration dynamics in movies of at least 12 h (725 min) for all three genetic MADM paradigms. We divided the developing cortical wall into two (lower and upper) zones. The lower zone comprised the VZ/SVZ and IZ, whereas the upper zone corresponded to the emerging CP. To empower our analysis, we employed a semi-automated tracking method to eliminate any experimenter bias (see Materials and Methods). We focused the analysis on the following four populations of MADM-labeled cells: control (red and green neurons in control-MADM), mosaic-control (red control neurons in *Cdk5r1*-MADM), mosaic-mutant (green *Cdk5r1*^−/−^ mutant neurons in *Cdk5r1*-MADM) and KO-mutant (red and green *Cdk5r1*^−/−^ mutant neurons in KO-*Cdk5r1*-MADM). First, we evaluated the relative distribution of cells with distinct genotypes across the two (upper and lower) zones at the start (t = 0) and the end (t = 725 min) of the recorded time series (Fig. 3E and F). We noticed that at the start time point roughly 50% of control, mosaic-control and KO-mutant (*Cdk5r1*^−/−^) cells were located in the upper and ~ 50% in the lower zone, respectively. In contrast, mosaic-mutant (*Cdk5r1*^−/−^) cells in *Cdk5r1*-MADM failed to distribute evenly between upper and lower zones, and accumulated significantly in the lower zone with much less cells in the upper zone. These data demonstrate that the cell-autonomous *Cdk5r1* function, to promote the transition from the IZ into the CP (e.g. Fig. 2B, F and J), is preserved in our *in vitro* migration assay. At the end time point, there was a trend whereby control and mosaic-control were slightly overrepresented in the upper zone relative to the amount of cells in the lower zone. However, mosaic-mutant neurons still displayed strong bias towards the lower zone, and KO-mutant cells remained at ~50/50 distribution at the end versus start time point.

Next we assessed migration speed (Fig. 3G) and directionality (Fig. 3H). Control and mosaic-control neurons did migrate at equal speeds in the upper and lower zones while mosaic-mutant and KO-mutant migrated at significantly slower speed in both compartments. Interestingly, KO-mutant neurons migrated even significantly slower than mosaic-mutant neurons in the lower zone. While control and mosaic-control neurons showed highly directional (vertical orientation from VZ toward pial surface) migration behavior in both, the upper and lower zones, mosaic-mutant and KO-mutant cells in the upper zone showed significantly less directional movement. In the lower zone, mosaic-mutant cells were not affected in directional movement when compared to control cells but migrating KO-mutant neurons showed significantly less directionality. Altogether, KO-mutant neurons migrated significantly slower and showed significantly less directional movement in the lower zone when compared to mosaic-mutant neurons. Thus, while both populations were *Cdk5r1*^−/−^, their environments were distinct (i.e. *Cdk5r1*^−/−^ only in KO-mutant). We therefore conclude that dynamics and directionality of radially migrating cortical projection neurons is critical dependent on the genetic landscape of the cellular environment.

### *In silico* modelling reveals cell-autonomous force generation and directionality in concert with environmental tissue resistance as minimal parameters instructing cortical projection neuron migration

**Figure 4:**
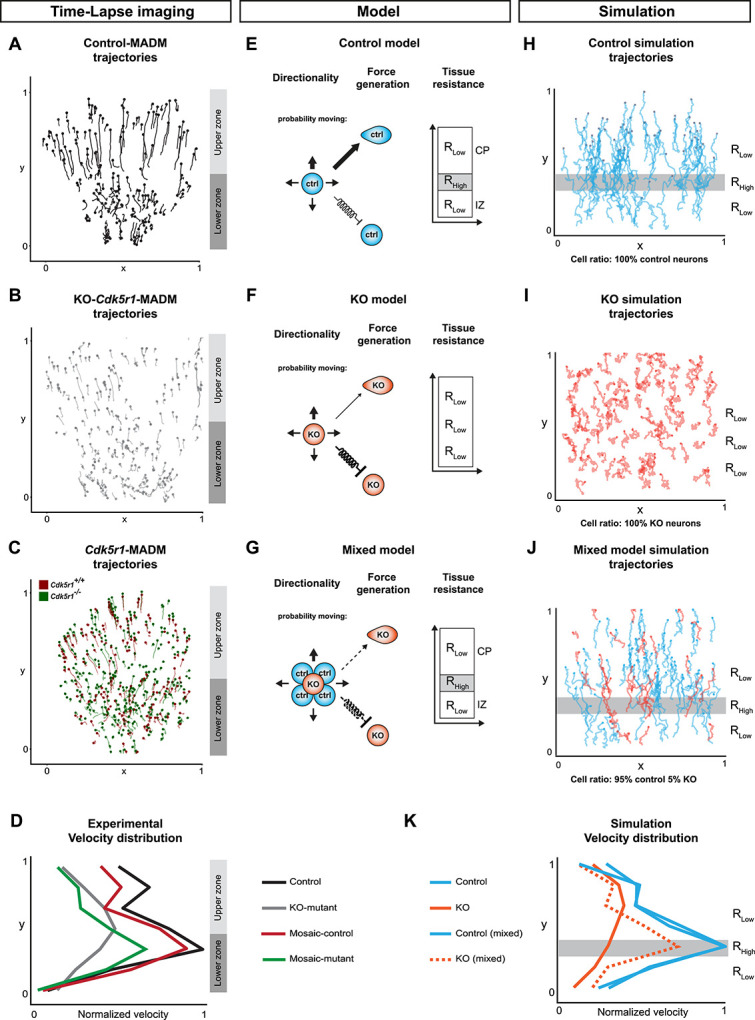
*In silico* modelling of neuronal migration dynamics upon MADM-based *Cdk5r1* ablation. (**A–C**) Representative migration trajectories in (A) control-MADM, (B) KO-*Cdk5r1*-MADM and (C) *Cdk5r1*-MADM. (**D**) Normalized velocity distributions from experimental data in control-MADM (n = 4), KO-*Cdk5r1*-MADM (n = 3) and *Cdk5r1*-MADM (n = 3). (**E**) Model of neuron migration in control environment with corresponding resistance zones. The thickness of the arrows indicates the probability to move in any direction. The random walk bias of cells in form of directionality and force generation when cells move (arrow) and with force conservation included as a spring constant when cells do not move (spring arrow). The directionality bias is defined as 65% in pial-direction for control. (**F**) Model of neuron migration in global *Cdk5r1* KO environment with a single resistance zone. The thickness of the arrows indicates the probability to move in any direction, here the directionality bias is defined as 51% in pial-direction. The random walk bias of cells in form of directionality and force generation when cells move (arrow) and with force conservation included as a spring constant when cells do not move (spring arrow). (**G**) Mixed model indicating cross interactions of *Cdk5r1^−/−^* mutant and control cells, respectively. The directionality bias is defined in pial-direction as a function of N_ctrl/N_Mut with a minimum at 51% and a maximum at 65%. The thickness of the arrows indicates the probability of the mutant neuron to move in any direction. The random walk bias of cells in form of directionality and force generation when cells move (arrow) and with force conservation included as a spring constant when cells do not move (spring arrow). (**H–I**) Simulation of migration trajectories in (H) control model, (I) global *Cdk5r1* KO model and (J) mixed model (95% Ctrl, 5% KO). Resistant zones are indicated accordingly. (**K**) Normalized velocity distributions of simulation trajectories.

The above data clearly demonstrated that the interplay of cell-autonomous gene function with non-cell-autonomous tissue-wide properties controls overall efficiency of cortical projection neuron migration. In order to obtain a more quantitative model we set out to define a minimal set of physical parameters sufficient to encompass migration dynamics on a statistical level. We based our approach on the cell tracking data, which we extracted from the time-lapse imaging experiments described above. We first analysed the trajectories of control cells from control-MADM (Fig. 4A), KO-mutant cells from KO-*Cdk5r1*-MADM (Fig. 4B) and mosaic-control and mosaic-mutant cells from *Cdk5r1*-MADM (Fig. 4C) and plotted the overall experimental velocity distribution of each cell population as a reference (Fig. 4D). More specifically, the temporal change in position resulted in a distribution of velocities (i.e. normalized velocity) throughout the developing cortical wall ranging from the ventricle to the pia (y axis in Fig. 4D). For control, mosaic-control and to some extent mosaic-mutant (cells in CP considered as ‘escapers’ being able to cross the IZ/CP border despite loss of *Cdk5r1*), neurons the distribution showed strong velocity peak within the lower half of the tissue (lower zone) followed by a sharp decrease in velocity around the border of the upper zone. However, KO-mutant cells did not display such characteristics but rather showed a more or less even distribution throughout the tissue. The velocity distributions and thus migration dynamics at the IZ-CP (lower–upper zone) border with a positional change in velocity implied a change in tissue architecture and suggested a difference in ‘stiffness’ between the two compartments. Indeed, atomic force microscopy measurements (i.e. Young’s moduli *E* as proxy for stiffness or resistance) previously indicated distinct stiffness of individual cortical compartments at different developmental stages [35].

**Figure 5:**
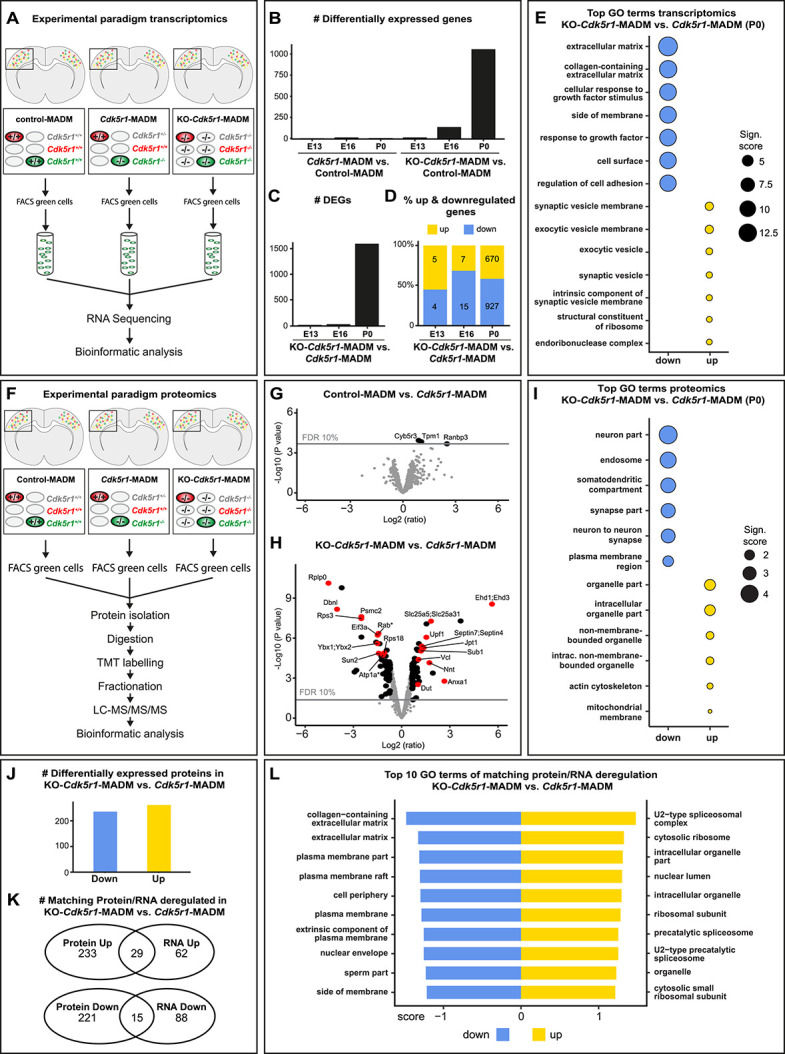
Gene and protein expression in *Cdk5r1^−/−^* mutant cells upon sparse and global KO. (**A**) Experimental paradigm and pipelines for gene expression profiling in control-MADM (left), *Cdk5r1*-MADM (middle) and KO-*Cdk5r1*-MADM (right). (**B**) Number of differentially expressed genes (DEGs) in *Cdk5r1*-MADM and KO*-Cdk5r1*-MADM versus control at E13, E16 and P0. (**C**) Number of DEGs in KO*-Cdk5r1*-MADM versus *Cdk5r1-*MADM at E13, E16 and P0. (**D**) Percentage of up- and downregulated genes in KO*-Cdk5r1*-MADM versus *Cdk5r1-*MADM at E13, E16 and P0. (**E**) Top GO terms associated with genes in (C and D) at P0. Note that GO term enrichments for upregulated genes are non-significant. (**F**) Experimental paradigm and pipelines for proteome profiling in control-MADM (left), *Cdk5r1*-MADM (middle) and KO-*Cdk5r1*-MADM (right). (**G**) Volcano plot showing deregulated proteins in control-MADM versus *Cdk5r1*-MADM comparison at P0. Note that only three proteins were significantly upregulated. (**H**) Volcano plot showing deregulated proteins KO*-Cdk5r1*-MADM versus *Cdk5r1*-MADM comparison at P0. Asterisks indicate that Rab and Atp1a protein groups consist of several isoforms not listed in the figure. (**I**) Top enriched GO terms associated with genes encoding the proteins as shown in (H). (**J**) Number of genes associated with differentially expressed proteins in KO-*Cdk5r1*-MADM versus *Cdk5r1*-MADM. Note that criteria for significant differential expression were relaxed compared to (H). (**K**) Venn diagrams indicating the overlap of deregulated genes in transcriptomic and proteomic datasets in KO-*Cdk5r1*-MADM versus *Cdk5r1*-MADM. (**L**) Top 10 GO-terms associated with gene sets that are up- and downregulated in both (transcriptomic and proteomic) data sets (overlap in K).

Next, from the experimental data we extracted two cell-intrinsic parameters, directionality (*ρ*) and force generation (*α*), and inferred one extrinsic parameter defining the environmental tissue resistance (*R_i_*) (Fig. 4E, see also Materials and Methods). For the latter, in a reductionist model, we assumed that IZ and CP are relatively homogenous and that overall stiffness can be attributed to differences in physical pore-size distribution. In other words, when a cell migrates through a porous environment it will have to squeeze through different pores in order to advance. Thus, the overall resistance that the cells are experiencing decreases with increasing pore size. The magnitude of the resistance fields *R_i_* is defined as a free parameter of our system. As described above, in control conditions, migrating cells at the IZ-CP border seem to struggle in order to squeeze through. In contrast, due to virtual inexistence of physiological layer structure in KO-*Cdk5r1*-MADM the migration dynamics appear independent of the location and thus of the *R_tissue_*. To integrate the two described environments in the model, we designed an environment with three compartments and two different resistances (*R_low_* and *R_high_*). In the control environment, the layers were defined by *R_Control,tissue_* = *R_1_*,*R_2_*,*R_3_* for *R_2_* > *R_1_* ~ *R_3_*, with *R_2_* roughly corresponding to the IZ-CP border (Fig. 5E and Table S2) while in KO-*Cdk5r1*-MADM environment we set *R_KO,tissue_* = *R_KO_* with magnitude on the order of *R_1_*,*R_3_* (Fig. 5F and Table S2). The tissue resistance parameters in combination with directional bias *ρ* and force generation *α* allowed us to model cells as persistent random walkers (Fig. S4A, see also Materials and Methods for details).

Based on our model, we next simulated control neuron migration (Fig. 4H) and could very closely mimic the experimentally observed migration behavior and velocity distribution (Fig. 4A, D, H and K). Likewise, the simulation of KO-mutant cells (Fig. 4I) in a uniformly low resistance environment *R_KO,tissue_* (with reduced directionality parameter *ρ* and force scaling parameter *α*, respectively) matched our observations in experimental data from KO-*Cdk5r1*-MADM paradigm (Fig. 4B, D, I and K).

Next we utilized a ‘mixed model’ where we introduced KO-mutant cells into a surrounding of control cells with according control tissue resistance environment (Fig. 4G). Here we introduced a linear coupling of directionality *ρ* and force generation coefficient *α* with the ratio of control to KO-mutant cells (Fig. 4J, Fig. S4B). The simulation conducted with the mixed model mimicked the dynamics and distributions we detected in *Cdk5r1*-MADM where mutant neurons appeared more dynamic than in KO-*Cdk5r1*-MADM (Fig. 4J–K). We could model the emergence of the non-cell-autonomous effects with increasing ratio of KO-mutant cells to control (Fig. S4C). The more KO-mutant cells present in the simulation, the more uniform the velocity distribution appeared across the vertical axis of the cortical wall. At a ratio of 4/96% (KO-mutant to control), the KO-mutant cells showed velocity distribution like mosaic-mutant cells and control cells (similar to the observation in the experimental data). In contrast, at a ratio of 10/90%, the KO-mutant cells displayed velocity distribution as observed in KO-*Cdk5r1*-MADM. Moreover, in our model, we found that the transition between the two distributions appeared abruptly between 5% and 6% abundance of KO-mutant cells. In summary, our *in silico* model can provide an accurate quantitative replicate of the migration behavior and dynamics as measured *in situ*. Our data further demonstrate that the relative amount of *Cdk5r1^−/−^* mutant cells and thus the overall genetic landscape within the cortical tissue critically impacts on the migration dynamics of individual cells.

### **Distinct deregulation of gene expression in *Cdk5r1*** ^***−/−***^ **mutant cells upon sparse and global KO**

Our data so far indicate distinct single-cell *in vivo* phenotypes in *Cdk5r1^−/−^* mutant cells depending on the genetic state of the environment. By using *in silico* modelling, we also showed that the tissue resistance (in combination with cell-intrinsic directionality and force generation capability) along the migration path represented a most critical physical parameter. To obtain a hint on molecular correlates mirroring the distinct observed phenotypes, we next pursued transcriptome analysis. We specifically isolated green GFP^+^ MADM-labeled cortical projection neurons by FACS [45, 46] in control-MADM (*Cdk5r1^+/+^*), *Cdk5r1*-MADM (*Cdk5r1^−/−^*) and KO-*Cdk5r1*-MADM (*Cdk5r1^−/−^*) at E13, E16 and P0, respectively (Fig. 5A). We performed RNA-sequencing of small bulk samples using SMARTer technology, followed by bioinformatics analysis (see Materials and Methods). First we analysed *Cdk5r1* expression and found that in both (sparse and global KO) deletion paradigms, the *Cdk5r1* expression level was close to zero while in control (*Cdk5r1^+/+^*) cells substantial *Cdk5r1* expression was evident (Fig. S5). These data validated the MADM-based *Cdk5r1* ablation paradigms. We next analysed differentially expressed genes (DEGs) in *Cdk5r1*-MADM and KO-*Cdk5r1*-MADM in comparison to control-MADM (Fig. 5B). At P0, we identified three significant DEGs in *Cdk5r1*-MADM, which was in stark contrast to the KO-*Cdk5r1*-MADM cells where we identified 1056 DEGs (padj<0.05, DESeq2). Thus depending on the state of the genetic environment (global *Cdk5r1* KO or not), three orders of magnitude higher number of DEGs was observed in individual *Cdk5r1^−/−^* mutant cells. Next, we directly compared the difference in DEGs between *Cdk5r1^−/−^* mutant cells in sparse versus global KO. We observed a progressive increase of DEGs during development with slightly more genes showing downregulation (Fig. 5C–D). To obtain more insight into the biological DEG functions we performed gene ontology (GO) enrichment analysis at P0 on the 670 up- and 927 downregulated genes (Fig. 5E). The top enriched GO terms for the downregulated genes (blue) were highly significant (padj<2.45x10^−9^, hypergeometric test) and associated with extracellular matrix (ECM), cell membrane and cell adhesion. GO term enrichment in the upregulated (yellow) genes was not significant (padj>0.5) and associated with synaptic terms (Fig. 5E). In summary, transcriptome analysis identified downregulation of ECM, membrane associated and cell adhesion genes as major classes of DEGs in *Cdk5r1^−/−^* cells in global KO-*Cdk5r1*-MADM but not sparse mosaic *Cdk5r1*-MADM.

### **Deregulation of ECM and cell adhesion proteins upon global but not sparse *Cdk5r1*** ^***−/−***^ **KO**

Next, we determined putative differences in *Cdk5r1^−/−^* mutant cells upon global versus sparse KO at the proteomic level. We thus isolated the green GFP^+^ MADM-labeled cortical projection neurons by FACS [45, 46] in control-MADM (*Cdk5r1^+/+^*), *Cdk5r1*-MADM (*Cdk5r1^−/−^*) and KO-*Cdk5r1*-MADM (*Cdk5r1^−/−^*) (Fig. 5F), followed by liquid chromatography–tandem mass spectrometry (LC–MS/MS) (Fig. 5F, see also Materials and Methods). First we compared the proteome of *Cdk5r1*-MADM mutant neurons to control-MADM cells whereby we identified (only) three significantly deregulated proteins (Fig. 5G). However, when mutant cells in *Cdk5r1*-MADM (*Cdk5r1^−/−^*) were compared with mutant cells in KO-*Cdk5r1*-MADM (*Cdk5r1^−/−^*), we identified 59 and 61 significantly up- and downregulated proteins, respectively (Fig. 5H). GO-term enrichment analysis (of the genes associated with the above differentially expressed proteins) indicated top GO terms associated with membrane and neuron–neuron contact among the downregulated group, thus corroborating our findings from the transcriptome analysis. GO-terms for the upregulated group were mainly associated with intracellular processes such as organelles (Fig. 5I). Having proteomic and transcriptomic information at hand, we next analysed putative overlap in the two data sets. Since the direct correlation between transcriptome and proteome was complex, we did lower the thresholds for differential expression (see Materials and Methods). We focused the analysis on 1060 gene annotations, which were informative in both data sets, and identified 262 downregulated and 236 upregulated DEGs from the proteomics data set (Fig. 5J). Strikingly, 29 upregulated and 15 downregulated genes were common to both transcriptomic and proteomic analyses (Fig. 5K). GO-term enrichment analysis identified ECM and membrane-associated GO terms among the down regulated gene group and GO terms related to cell-intrinsic entities among the upregulated gene group (Fig. 5L). In summary, the above analysis revealed significant downregulation of mRNAs and proteins related to membrane, cell adhesion and ECM, specifically in *Cdk5r1^−/−^* mutant cells upon global tissue-wide but not sparse KO.

### Global tissue-wide effects predominates the cell-autonomous phenotype due to loss of *Dab1*

**Figure 6:**
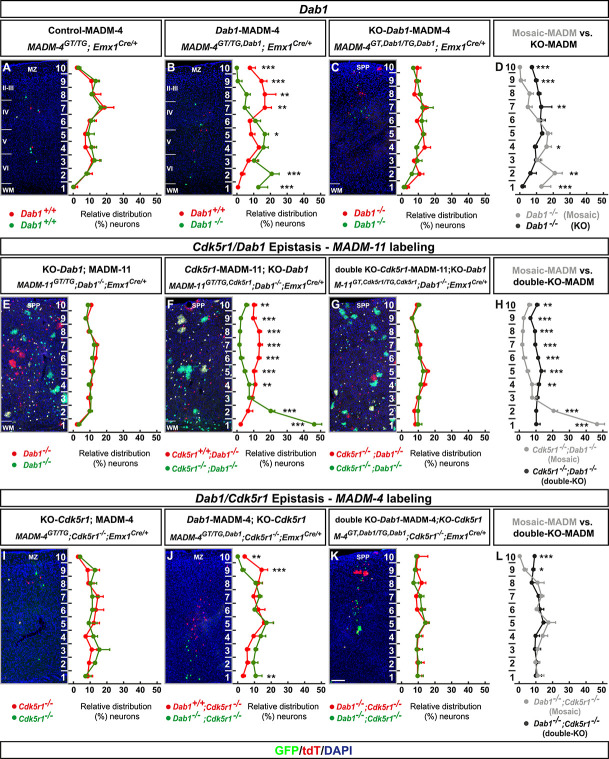
MADM-based analysis of *Dab1* and *Cdk5r1*/*Dab1* epistasis. (**A–D**) Analysis of green (GFP^+^) and red (tdT^+^) MADM-labeled projection neurons in (a) control-MADM (*MADM-4^GT/TG^;Emx1^Cre/+^*); (F) *Dab1*-MADM (*MADM-4^GT/TG,Dab1^;Emx1^Cre/+^*); and (G) KO-*Dab1*-MADM (*MADM-4^GT,Dab1/TG,Dab1^;Emx1^Cre/+^*). Relative distribution (%) of MADM-labeled projection neurons is plotted in 10 equal zones across the cortical wall. (H) Direct distribution comparison of *Dab1^−/−^* mutant cells in *Dab1*-MADM (grey) versus KO-*Dab1*-MADM (black) distribution. (**E–H**) *Cdk5r1*/*Dab1* epistasis in MADM-11 labeling background. Analysis of green (GFP^+^) and red (tdT^+^) MADM-labeled projection neurons in (E) KO-*Dab1*; MADM-11 (*MADM-11^GT/TG^;Dab1^−/−^;Emx1^Cre/+^*), (F) *Cdk5r1*-MADM-11;KO-*Dab1* (*MADM-11^GT/TG,Cdk5r1^;Dab1^−/−^;Emx1^Cre/+^*) and (G) double-KO-*Cdk5r1*-MADM-11;KO-*Dab1* (*MADM-11^GT,Cdk5r1/TG,Cdk5r1^;Dab1^−/−^;Emx1^Cre/+^*). Relative distribution (%) of MADM-labeled projection neurons is plotted in 10 equal zones across the cortical wall. (H) Direct distribution comparison of *Cdk5r1^−/−^* mutant cells upon sparse (grey) and global (black) KO in *Dab1^−/−^* mutant background. (**I–L**) *Dab1/Cdk5r1* epistasis in MADM-4 labeling background. Analysis of green (GFP^+^) and red (tdT^+^) MADM-labeled projection neurons in (I) KO-*Cdk5r1*; MADM-4 (*MADM-4^GT/TG^;Cdk5^−/−^;Emx1^Cre/+^*), (J) *Dab1*-MADM-4;KO-*Cdk5r1* (*MADM-4^GT/TG,Dab1^;Cdk5r1^−/−^;Emx1^Cre/+^*) and (G) double-KO *Dab1*-MADM-4;KO-*Cdk5r1* (*MADM-4^GT,Dab1/TG,Dab1^;Cdk5r1^−/−^;Emx1^Cre/+^*). Relative distribution (%) of MADM-labeled projection neurons is plotted in 10 equal zones across the cortical wall. (H) Direct distribution comparison of *Dab1^−/−^* mutant cells upon sparse (grey) and global (black) KO in *Cdk5r1^−/−^* mutant background. All analysis was carried out at P21. N = 3 for each genotype. From each animal 10 (MADM-11) or 20 (MADM-4) hemispheres were analysed. Nuclei were stained using DAPI (blue). Data indicate mean ± SD, ^*^P < 0.05, ^**^P < 0.01 and ^***^P < 0.001. Marginal zone (MZ), superplate (SPP), white matter (WM). Scale bar: 100 μm.

Global elimination of p35/CDK5 signaling in cortical projection neurons results in dominant tissue-wide effects that affect the individual cell-autonomous phenotype. To evaluate whether such observation is specific to p35/CDK5 signaling pathway we next investigated the consequences of ablation of Reelin/DAB1 signaling (which operates in parallel to CDK5 [7, 39, 44, 59]). We therefore generated MADM-based *Dab1* (located on chr.4) ablation paradigms using MADM-4 platform (Fig. S2C). We analysed radial neuron migration in control-MADM (*MADM-4^GT/TG^;Emx1^Cre/+^*), *Dab1*-MADM (*MADM-4^GT/TG,Dab1^;Emx1^Cre/+^*) and KO-*Dab1*-MADM (*MADM-4^GT,Dab1/TG,Dab1^;Emx1^Cre/+^*) at E16, P0 and P21 in somatosensory cortex (Figs 6A–D and S6). In *Dab1*-MADM the majority of *Dab1^−/−^* mutant neurons were positioned in a biased manner, reflecting the cell-autonomous *Dab1* function [18, 24, 68] within lower zones in the distribution charts (i.e. below CP during earlier development and lower cortical layers at later stages). Conversely, *Dab1^−/−^* mutant neurons in KO-*Dab1*-MADM showed much more even distribution throughout the cortical wall (Figs 6A–D and S6).

### Tissue-wide non-cell-autonomous effects impacting neuronal migration are specific for distinct signaling pathways

Global, but not sparse KO, of *Dab1* results in tissue-wide effects affecting the single-cell *Dab1^−/−^* mutant phenotype in quite a similar manner to the above p35/CDK5 findings. Whether the observed tissue-wide effects exhibit ‘gene’ specificity or may dominate over distinct signaling pathways was however not clear. We thus conceived epistasis experiments (Fig. S7) to test possible pathway exclusivity of tissue-wide properties upon global gene KO. First, we utilized MADM-11 platform to generate control, global and sparse *Cdk5r1* deletion paradigms in a full *Dab1^−/−^* KO background (Fig. 6F–H). We thus tested whether tissue-wide effects due to global *Dab1* ablation may affect or interfere with the cell-autonomous *Cdk5r1^−/−^* mutant phenotype (i.e. accumulation of mutant cells in lower layers and the white matter as described in Fig. 1F). We however observed a similar cell-autonomous *Cdk5r1^−/−^* mutant phenotype (upon sparse mosaic *Cdk5r1* deletion) in *Dab1^−/−^* background (Fig. 6F) as in *Dab1^+/+^* background (i.e. in *Cdk5r1*-MADM context). Next we reversed the genetic background conditions and utilized MADM-4 platform to generate control, global and sparse *Dab1* deletion paradigms in a full *Cdk5r1^−/−^* KO background (Fig. 6I–L). Again, the cell-autonomous *Dab1^−/−^* mutant phenotype upon sparse deletion was highly similar in *Cdk5r1^−/−^* KO (Fig. 6J) when compared to *Dab1*-MADM in *Cdk5r1^+/+^* background (Fig. 6B). In contrast to the above findings, concomitant global KO of both *Cdk5r1* and *Dab1* resulted in a relatively uniform distribution of double mutant cells across the cortical wall (Fig. 6G and K), comparable to the distribution in individual KO of *Dab1* (Fig. 6E) or *Cdk5r1* (Fig. 6I), respectively. In summary, tissue-wide non-cell-autonomous effects predominates the cell-autonomous phenotype albeit in a gene-specific manner.

### Global KO of both *Cdk5r1* and *Dab1* triggers deregulation of genes associated with ECM and cell adhesion

**Figure 7:**
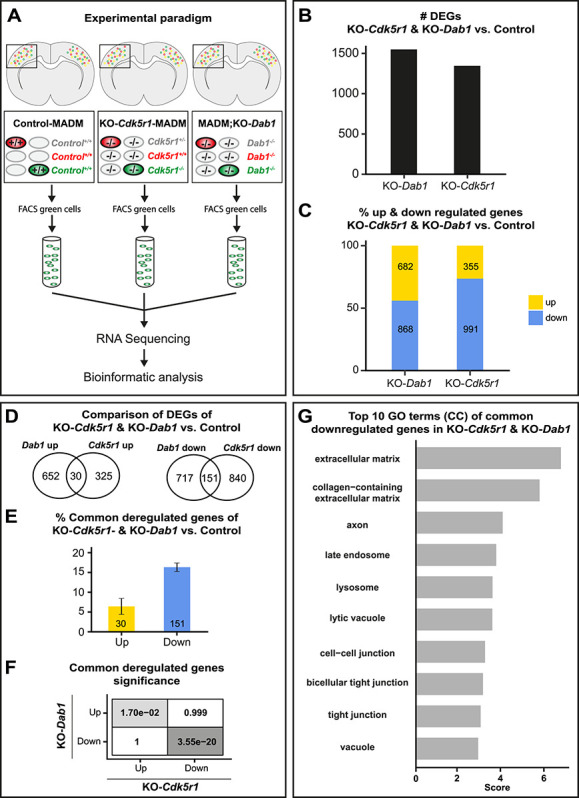
Gene expression upon combined global KO of *Cdk5r1* and *Dab1.* (**A**) Experimental paradigm and pipelines for gene expression profiling in control-MADM (left), KO-*Cdk5r1*-MADM (middle; KO-*Cdk5r1*) and MADM;KO-*Dab1* (right; KO-*Dab1*) at P0. (**B**) Number of differentially expressed genes (DEGs) in KO-*Cdk5r1* and KO-*Dab1* versus control. (**C**) Percentage of up- and downregulated genes in KO-*Cdk5r1* and KO-*Dab1* versus control. (**D**) Venn diagrams showing common up- and down-regulated genes in KO-*Cdk5r1* and KO-*Dab1* versus control. (**E**) Percentage of common up- and downregulated genes in KO-*Cdk5r1* and KO-*Dab1* versus control. (**F**) Significance of all pairwise overlaps of DEGs shown in (D). (**G**) Top 10 GO terms of commonly downregulated genes in KO-*Cdkr5r1* and KO-*Dab1*, according to overlap shown in (D, right). Commonly upregulated genes did not yield any significant GO term enrichment.

The phenotypic manifestation of global tissue-wide effects appeared very similar for *Cdk5r1* and *Dab1* because global ablation of either gene resulted in relatively uniform distribution of mutant neurons across the vertical axis of the cortical wall. We therefore asked next whether we might find overlap in deregulated gene expression upon global *Cdk5r1* and *Dab1* LOF or not. To this end, we applied the identical approach as described above and FACS-isolated green GFP^+^ MADM-labeled cells (using MADM-11 platform) from control-MADM (*MADM-11^GT/TG^;Emx1^Cre/+^*) and MADM;KO-*Dab1* (*MADM-11^GT/TG^;Dab1^−/−^;Emx1^Cre/+^*) at P0 (Fig. 7A). We complemented this dataset with control-MADM (*MADM-11^GT/TG^;Emx1^Cre/+^*) and KO-*Cdk5r1*-MADM (*MADM-11^GT,Cdk5r1/TG,Cdk5r1^;Emx1^Cre/+^*) data as described before (Fig. 5). We performed DEG analysis on the combined data set (see also Materials and Methods). *Dab1* expression level was close to zero in MADM;KO-*Dab1* but readily detectable in all other samples, validating the MADM;KO*-Dab1* ablation paradigm (Fig. S8A).

Next, we analysed DEGs in KO-*Cdk5r1-*MADM and MADM;KO-*Dab1*, relative to control, and compared the data to each other. Upon global ablation of either, *Cdk5r1* or *Dab1*, we found >1000 deregulated genes in both mutants (Fig. 7B) with the majority of DEGs being downregulated (Fig. 7C). We next analysed the overlap of DEGs in *Cdk5r1* and *Dab1* KOs and found both common and non-common deregulated genes (Fig. 7D). The majority of the commonly deregulated genes were downregulated (Fig. 7E) and the overlap of both, up- and downregulation was significant (Fig. 7F). Among the upregulated genes we did not obtain any significantly enriched GO-terms although the non-common deregulated genes showed overlap of many of their respective GO-terms between KO-*Cdk5r1-*MADM and MADM;KO-*Dab1* (Fig. S8B). In contrast, among the commonly downregulated genes, we found significant GO-terms associated with cell–cell and cell–matrix interaction (Fig. 7G). Taken together, the above analysis suggested that cell-adhesion and ECM represent major elements in tissue-wide effects upon global KO of genes encoding components of the p35/CDK5 and Reelin/DAB1 signaling pathways.

## DISCUSSION

**Figure 8:**
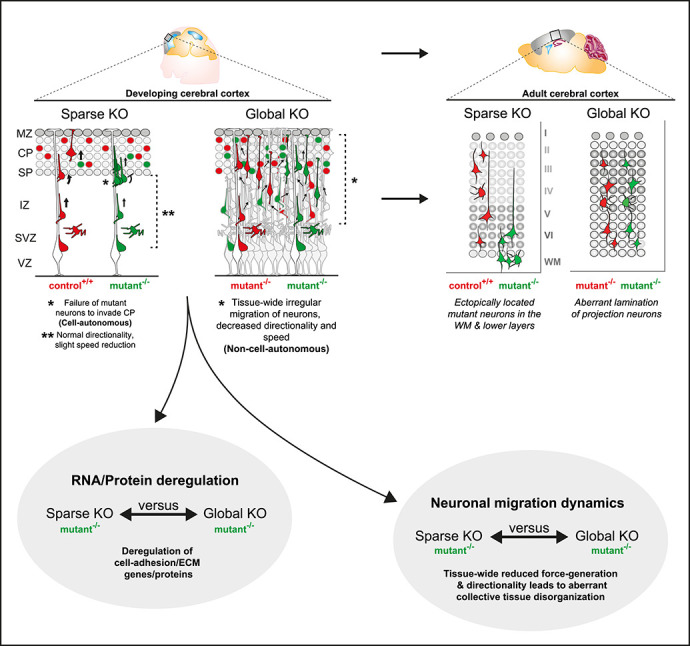
Interplay of cell-autonomous and global tissue-wide properties in cortical projection neuron migration. Schematic illustrating the MADM-based subtractive phenotypic analysis of sparse genetic mosaics (control background) and global knockout (cKO/KO) (mutant background), both coupled with fluorescent MADM-labeling of homozygous mutant and control neurons. Such assay enabled the high-resolution analysis of projection neuron migration dynamics in distinct genetic environments with concomitant isolation of genomic and proteomic profiles. In combination with computational modeling, we utilized these experimental paradigms to visualize non-cell-autonomous effects in radial neuron migration at single-cell resolution. In sparse KO, mutant neurons migrated more dynamically and expressed cell adhesion molecules similar like in control. However, in global KO, we observed that cell adhesion molecules were significantly downregulated. Mutant neurons in global KO also showed much more severe migration phenotype resulting in drastic disorganization of the mature cortical wall.

Radial migration of cortical projection neurons has been studied extensively, and a rich catalogue of regulatory signaling cues and pathways have been compiled. The systematic study of the cell-autonomous functions of the genes encoding the signaling cascades has revealed an extensive genetic framework. However, the nature and relative contributions of global tissue-wide effects, in regulating radial migration in the developing neocortex, remained unclear. To this end, we established genetic paradigms enabling the dissection of cell-autonomous gene function and non-cell-autonomous effects. Our data revealed that the genetic landscape of the cellular environment, surrounding migrating neurons, fundamentally affects the individual single-cell phenotype (Fig. 8).

We focused our analysis mainly on the well-characterized p35/CDK5 pathway providing a defined genetic framework and context. We could show that sparse p35/CDK5 ablation results in a highly specific phenotype that we attributed to the loss of cell-autonomous gene function. Individual *p35/Cdk5^−/−^* mutant cortical projection neurons in sparse genetic mosaic conditions failed to pass the border between the IZ and the emerging CP and progressively accumulated below the CP and the white matter. However, radial migration from the VZ and through the IZ occurred, albeit at lower speed. In contrast, single *p35/Cdk5^−/−^* mutant neurons in a global homozygous *p35/Cdk5^−/−^* mutant KO environment migrated even slower in the VZ/IZ and showed skewed directionality. The perhaps more surprising finding was that in the above full KO condition, *p35/Cdk5^−/−^* mutant neurons distributed relatively evenly across the entire cortical wall. Thus, the cell-autonomous phenotype as observed in sparse KO scenario seemed wiped out. There are a few (not mutually exclusive) possibilities that could explain the distinct phenotypic observations. First, the IZ-CP border lost its property as a physical gatekeeper upon global loss of *p35/Cdk5*. Such scenario actually formed an important basis of our modeling approach where we assumed uniform tissue stiffness/resistance that radially migrating neurons encounter upon global p35 ablation. Indeed, the simulation of such conditions revealed very similar migration trajectories of *p35/Cdk5^−/−^* mutant cells as observed by live imaging in experimental global *p35/Cdk5* KO conditions. Second, *p35/Cdk5^−/−^* mutant neurons in sparse genetic mosaic lost an essential intrinsic property required for transiting the IZ-CP border, reflecting the cell-autonomous *p35/Cdk5* gene functions. One important p35/CDK5 downstream signaling hub is NDEL1, which is phosphorylated at specific sites by CDK5 [56, 69]. Interestingly, sparse mosaic deletion of *Ndel1* results in a congeneric phenotype, to the sparse *p35/Cdk5^−/−^* condition, whereby *Ndel1^−/−^* mutant neurons fail to invade the CP and progressively accumulate below the CP and later in the white matter [32]. However, tissue-wide *Ndel1* KO results in severely disorganized neocortex with seemingly immobile *Ndel1^−/−^* mutant neurons [84]. Thus, sparse loss of any component of the p35/CDK5/NDEL1 signaling hub leads to the inability of individual radially migrating neurons to enter the target area, the emerging CP, while global KO results in predominant cortical tissue disorganization with slower or even immobile projection neurons. The sparse ablation of p35/CDK5/NDEL1 might lead to deficits in LIS1/Dynein-mediated force generation [8, 36, 51, 65, 81], necessary to overcome the increased physical tissue resistance at the IZ/CP border, although the precise biochemical mechanism remains to be clarified in future studies.

The observation that global KO, of the same signaling component as in sparse KO, results in predominant tissue-wide effects appears to exhibit pathway specificity. As such, sparse elimination of *Cdk5r1* gene function in a *Dab1^−/−^* global KO background (which also shows severe disorganization of the cortical wall) showed a similar cell-autonomous phenotype (failure to enter CP) like in *Dab1^+/+^* background conditions. Thus, the cell-autonomous *Cdk5r1^−/−^* phenotype was preserved regardless of the global *Dab1* genotype. It will be revealing to investigate the generality of the above findings in future experiments including systematic assessment of potential epistatic interactions of mutations in distinct genes that lead to cortical malformation.

*Cdk5r1* and *Dab1* knockout mouse models show a difference in their neuronal layering distribution with *Dab1* mutants presenting inverted layering [68]. In contrast the *Cdk5r1* mutants show intermingled upper and lower layer neurons [11, 23] (Fig. S3). As the cell-autonomous *p35^−/−^* phenotype was preserved in the global *Dab1* environment, we suggest that the *p35^−/−^* phenotype arises due to the inability to surpass previously born neurons rather than due to a deficit in crossing the subplate. Along this line, a recent study showed that the density of deep layer *Dab1* deficient neurons prevented superficial layer neurons from entering the cortical plate [83]. Difference in tissue resistance/stiffness is an attractive explanation for the accumulation of the *Cdk5r1^−/−^* mutant cells in the *Dab1^−/−^* global KO background. For example, if the *Dab1^−/−^* KO environment is stiffer than the *Cdk5r1^−/−^* KO environment, passing cells will face a higher tissue resistance and their migration will be impaired. So far, biophysical measurement and data of tissue resistance in brain tissue are very limited. Future studies investigating such properties will undoubtedly provide important insights into our understanding of the gene mutations affecting neuronal migration.

What are the underlying characteristics of global tissue-wide effects affecting radial projection neuron migration? It is clear that any tissue consists of a complex extracellular environment. Thus, individual cells are exposed to many extrinsic elements including (i) secreted factors acting locally, globally or even systemically; (ii) the ECM; and (iii) neighboring cells mediating cell–cell interactions through receptors and/or direct physical stimuli [28]. While secreted factors mostly signal to other cells (besides autocrine signaling), and thus mainly act non-cell-autonomously, how cell-intrinsic signaling molecules could act at the global tissue-wide level is not yet clear. Our MADM data are based on the genetic deletion of the intracellular CDK5 kinase or DAB1 adapter protein function. In both instances, the cell-autonomous migration phenotype upon sparse deletion differed from the one upon global KO. In order to get insights at the molecular level, we pursued transcriptome/proteome analysis and found that upon sparse deletion of for instance p35, very few genes and proteins were deregulated in mutant cells. In stark contrast, global KO led to a high number of deregulated proteins and genes. A sizeable fraction of differentially expressed genes even correlated significantly with deregulation of their encoded proteins. The top GO terms associated with deregulated genes/proteins included ECM, receptors and cell adhesion. These results indicate that at the global tissue-wide level the ECM and cell adhesion landscape was drastically changed in global KO condition when compared to wildtype. Although p35/CDK5 and DAB1 signal in parallel, deregulated ECM and cell adhesion was a common denominator upon global KO. Our data are in agreement with earlier studies that showed N-cadherin dependent signaling to be relevant for both p35/CDK5 [42] and DAB1 [18, 71, 72] pathways, respectively. Interestingly, transcriptomic analysis in whole cortex KO mouse models for *Ndel1*, *Lis1* and *Ywhae*, all acting in the LIS1/Dynein signaling pathway and to some extent downstream of p35/CDK5, have also revealed altered cell adhesion and cytoskeleton organization pathways [63].

Migrating neurons can exert positive and negative interaction on each other depending on the cellular environment and their genetic constitution [19, 21, 28]. In collective cell migration for instance, cell–cell interaction balancing adhesion and repulsion is a key mechanism [73]. Besides, direct physical interactions might be critical whereby for example less agile mutant cells are passively pushed, pulled or simply piggyback onto more dynamic cells.

To better comprehend tissue-wide effects on neuronal migration, it will be important to investigate the migration behavior in combination with transcriptomic and proteomic profiles of rescued ‘wild type’ neurons in a global KO environment. A few studies have observed a rescue of migration by re-expression of the mutant gene in a subset of neurons in a global-KO of the same gene [23, 68, 74]. Examples include chimeric mice consisting of *Dab1* wild-type and *Dab1* mutant cells. In such *Dab1*-KO environment *Dab1* wild-type cells were capable of migrating radially but failed to rescue the inversion of cortical layers of the *Dab1*-KO. Interestingly, *Dab1* mutant cells did not impose a mutant phenotype on the wild-type cells [24]. Moreover, induction of *Dab1* expression in a subset of superficial layer neurons in *Dab1*-deficient mice using *in-utero* electroporation [55] or lentivirus vectors [68] rescued the migration phenotype of the transfected cells. Taken together, these findings suggest that the effect of a mutant-environment on rescued wild type cells is minimal although subtle changes in transcriptomics/proteomics cannot be excluded.

Our data indicate that ECM and/or cell–cell interaction are perturbed in *Dab1*-KO. Since sparse re-expression of *Dab1* rescues the migration capability [74] we propose that impaired tissue-wide ECM/cell–cell interaction did not predominantly impair the cell-autonomous migration capability of rescued cells. However, the single-cell *Dab1*-KO phenotype likely precipitated due to the interaction of impaired ECM/cell–cell environment (tissue-wide effect) with the *Dab1* deficient cell. Such scenario would be supported by our finding that *Dab1* deficient cells in a phenotypically normal *Dab1* environment (normal ECM/cell–cell interaction) showed a significantly different migration phenotype compared to *Dab1* deficient cells in a global *Dab1* KO environment.

Radial glia cells play an important role in neuronal migration and therefore the mutant radial glia could potentially contribute to the migration defect. However, multiple lines of evidence argue against this possibility. In the case of RGCs exhibiting a dominant effect on migrating neurons, phenotypes would appear throughout the cortical wall. In contrast to such expectation, we found that mutant neurons in mosaic-MADM and KO-MADM did migrate through the IZ and sometimes beyond into the CP. In addition, migration defects were strongest in the outer zones of the cortical wall. Studies re-expressing either *Cdk5r1* or *Dab1* specifically in a subset of neurons in a global-KO environment observed a ‘rescue’ of the targeted neurons in their ability to migrate, which suggest no substantial direct contribution of either *Cdk5r1* or *Dab1* mutant radial glia on the migrating neurons [23, 68, 74]. Detailed morphological analysis found no differences between wild-type and p35 KO RGPs [11].

Labelling efficiency did vary between the different MADMs [12] dependent on the chromosome and insertion. However, the phenotypes observed between *Cdk5r1*, *Cdk5* and *Dab1* did show a similar cell-autonomous versus non-cell-autonomous phenotype regardless of the MADM labelling efficiency. As such, the distribution pattern of the labeled neurons was highly similar when comparing the different control-MADM scenarios. Furthermore, only slight differences were observed between e.g. *Cdk5r1* (MADM-11) and *Cdk5* (MADM-5) for all MADM genotypes analysed. These differences were most likely due to individual gene (loss of) function rather than a reflection of the efficiency of the MADM reporter. However, we cannot completely exclude local effects due to more or less mutant cells in distinct MADM reporters.

MADM relies on interchromosomal recombination and differential segregation of recombinant chromosomes during mitotic cell division to generate homozygous mutant cells for a particular gene of interest. Thus, mutant cells that are generated during the MADM process could in principle inherit certain amounts of the corresponding protein during cell division, a process called protein perdurance. We cannot completely exclude a possible effect of protein perdurance in the interpretation of the phenotypes described in the above mosaic sparse deletion paradigms. However, we consider the effect to be minimal at best for the following reasons. The *Emx1*-Cre driver is active from E9 onward when neural stem cells in the mouse embryonic forebrain still divide in an expanding proliferative manner with relatively short cell-cycle length of ~8 h [77]. Thus, before the generation of postmitotic projection neurons, every proliferative stem cell division will dilute protein perdurance. Assuming that half the amount of protein from the mother cell was inherited into the daughter cells, after five cell cycles, <4% of protein would be left in the cells if the protein were infinitely stable. Yet, the p35 protein encoded by *Cdk5r1*, is a very short lived protein with a half-life of 20-30 min *in vivo* [60] and DAB1 has a half-life of ~12 h [3]. Hence, it is unlikely that a biologically relevant amount of p35 or DAB1 protein was present in a substantial number of mutant migrating cells in any of the mosaic MADM scenarios.

Our findings put a new perspective on the clinical symptoms and/or appearance of disease-causing mutations affecting radial migration in particular, but also mutations causing cortical malformation in general. For instance in focal malformations of cortical development (FMCD) a small fraction of mutant cells can disrupt neighboring cells and even large areas thereby affecting overall cortical architecture [5, 47, 62, 66]. In order to obtain a better understanding of FMCD disease etiology, it will thus be essential to rigorously scrutinize the contribution of not only cell-autonomous loss of gene function but also tissue-wide and systemic effectors. Interestingly, lissencephaly with cerebellar hypoplasia has been attributed to individuals with mutations in *CDK5* or *RELN* [33, 48]. The common clinical appearance in such patients could (at least in part) emerge from tissue-wide effects alike the ones we observed in our MADM-based analysis of *Cdk5r1/CDK5* and *Dab1*, respectively.

## CONCLUSION

Our study provides quantitative evidence, in a correlative manner, that global tissue-wide effects play essential roles in the control of radial projection neuron migration in the developing cortex. Based on defined genetic conditions in combination with single-cell tracing, we could show that sparse mosaic ablation of gene function results in highly specific migration phenotypes. In contrast, in global KO, individual migrating neurons exhibit distinct deficits that result from predominant tissue-wide effects. Altogether, cortical projection neuron migration is tightly regulated by intrinsic gene function and depending on the cellular and genetic landscape of the overall surrounding tissue.

## MATERIALS AND METHODS

### Mouse lines

All mouse colonies were maintained in accordance with protocols approved by the institutional animal care and use committee, institutional ethics committee and the preclinical core facility at Institute of Science and Technology Austria. Experiments were performed under a license approved by the Austrian Federal Ministry of Science and Research following the Austrian and EU animal laws (license numbers: BMWF-66.018/0007-II/3b/2012 and BMWFW-66.018/0006-WF/V/3b/2017).

Mice with specific pathogen-free status according to FELASA recommendations [50] were bred and maintained in experimental rodent facilities (room temperature 21 ± 1°C [mean ± SEM]; relative humidity 40%–55%; photoperiod 12 L:12D). Food (V1126, Ssniff Spezialitäten GmbH, Soest, Germany) and tap water were available ad libitum.

Mouse lines with MADM cassettes inserted on chr.4, chr.5 and chr.11 [12, 32] [MADM-4-GT, MADM-4-TG, MADM-5-GT, MADM-5-TG, MADM-11-GT (JAX stock # 013749), MADM-11-TG (JAX stock # 013751)], *Cdk5r1* [11] (JAX stock # 004163), *Cdk*5-flox [67] (JAX stock # 014156), *Dab1* [34] (JAX stock # 003581); *Emx1*-Cre [20] (JAX stock # 005628) were previously described. We have not observed any influence of sex on the results in our study, and all experiments and analyses were carried out using animals of both sexes. Phenotypic time course analysis of *Dab1*-MADM-4, *Cdk5*-MADM-5, *Cdk5r1*-MADM-11 in combination with *Emx1*-Cre was performed at E14, E16, P0 and P21. For sequencing and/or proteomics experiments, MADM-11 animals were used in combination with *Emx1*-Cre and were analysed at E13, E16 and P0. Genetic epistasis experiments of *Cdk5r1* and *Dab1* on MADM-4 and MADM-11 in combination with *Emx1*-Cre were all performed at P21.

### Isolation of fixed tissue

Tissues from postnatal time points (P15/P21) were collected by cardiac perfusion. Mice were deeply anesthetized through injection of a ketamine/xylazine/acepromazine solution (65 mg, 13 mg, and 2 mg/kg body weight, respectively) and unresponsiveness was confirmed through pinching the paw. The diaphragm of the mouse was opened from the abdominal side to expose the heart. Cardiac perfusion was performed with phosphate buffered saline (PBS) followed immediately by ice-cold 4% PFA prepared in PB buffer (Sigma-Aldrich). Brains were removed and further fixed in 4% PFA for 24 h at 4°C to ensure complete fixation. Brains were cryopreserved with 30% sucrose (Sigma-Aldrich) solution in PBS for ~48 h. Brains were then embedded in Tissue-Tek O.C.T. (Sakura). For adult time points, 45 μm coronal sections were collected in 24 multi-well dishes (Greiner Bio-one) and stored at −20°C in antifreeze solution (30% v/v ethylene glycol, 30% v/v glycerol, 10% v/v 0.244 M PO4 buffer) until used. Tissue from embryonic time points (E14/E16) and postnatal day zero (P0) was directly transferred into 4% PFA and kept at least 24 h at 4°C. Cryopreservation and embedding were done as described for adult brains. For embryonic and early postnatal brains, 25 μm cryosections were directly mounted onto Superfrost glass-slides (Thermo Fisher Scientific).

### Immunohistochemistry

Brain sections were mounted onto Superfrost glass-slides (Thermo Fisher Scientific) and let to dry, followed by three wash steps each of 5 min with PBS. Tissue sections were blocked for 30 min in a buffer solution containing 5% normal donkey serum (Thermo Fisher Scientific) and 0.5% Triton X-100 in PBS. Primary antibodies for GFP (1:500 Chick, Aves Labs Inc.) and RFP (1:500 Rabbit, MBL) were mixed in the blocking buffer and incubated on the tissue for at least 12 h at 4°C. Sections were washed 3 times for 5 min each with PBS with triton (PBT) (0.5% Triton X-100 in PBS) and incubated with the corresponding secondary antibodies Alexa Fluor 488 (1:500 Anti-Chicken IgG, Jackson ImmunoResearch Labs) and Cy3 (1:500 Anti-Rabbit IgG, Jackson ImmunoResearch Labs) diluted in PBT for 1 h. Sections were then washed two times with PBT and once with PBS each for 5 min. Finally, nuclear staining was done using 10 min of incubation with PBS containing 2.5% DAPI (Thermo Fisher Scientific). Sections were embedded in mounting medium containing 1,4-diazabicyclooctane (DABCO; Roth) and Mowiol 4-88 (Roth) and stored at 4°C. For the neuronal layer identity staining, primary antibodies for Cux1 (1:100, Goat, Santa Cruz), FoxP2 (1:300, Goat, Santa Cruz) and Ctip2 (1:400, Rat, Abcam) were used with primary antibodies for GFP (1:500, Chick, Aves Labs Inc.) and RFP (1:500, Rabbit, MBL) in the same manner as described above. Alexa Flour 647 (1:500 Anti-Goat IgG or 1:500, Anti-Rat IgG,Jackson ImmunoResearch Labs) were used for Goat or Rat originating primary antibodies. For primary antibodies requiring antigen retrieval, the tissue was processed by incubating in sodium citrate buffer (10 mM Sodium Citrate, 0.05% Tween 20, pH 6.0) at 80°C for 10 min prior to immunostaining. Once cooled to room temperature, sections were washed with PBS and standard immunostaining protocol as described above was performed.

### Imaging of fixed brain tissue

Histological brain sections were imaged using confocal microscopy (Zeiss inverted LSM800) or epifluorescence microscopy (Olympus VS120 Slide scanner). Confocal images were recorded on a Zeiss LSM 800 laser-scanning confocal microscope mounted with a plan-apochromat 10x/0.45, WD = 2.1 mm objective. Excitation/emission wavelengths were 488/509 nm (EGFP), 554/581 nm (tdTomato), and 353/465 nm (DAPI). Z-series images were collected on a PC running ZEN 2.6 software (Zeiss). Image series were Z-projected, stitched and contrast-enhanced using ZEN 2.6 software (Zeiss). Slidescanner images were recorded with a 10x/NA 0.4 objective. Excitation/emission wavelengths were 485/518 nm (FITCH), 560/580 nm (Cy3), and 387/455 nm (DAPI). Slide scanner images were processed using ImageJ software [70].

### Analysis of relative distribution of MADM-Labeled neurons

Images were imported into ImageJ software [70] and MADM-labeled neurons were manually quantified based on the respective fluorescent marker expression and their relative position, which was calculated with respect to the bottom of the ventricle and the pial surface (for details see http://github.com/hippenmeyerlab/cell2layer). The analysis script used computes the relative and absolute distances of each manually marked neuron to its boundaries (ventricular surface and pia) provided manually as two segmented lines. For each neuron, the shortest distance to the two-layer boundaries is computed, resulting in two distances d_1_ and d_2_. The normalized (relative) distance is computed by:

$$ relativedistance=\frac{d_1}{\left({d}_1+{d}_2\right)} $$

Statistical analyses were done with GraphPad Prism 8.0.1, applying an arcsin conversion of relative percentages, a two-way analysis of variance (ANOVA) and a Tukey post hoc test.

### Slice culture and time-lapse imaging

Embryos were collected at E16 and stored in ice-cold PBS during genotyping. Immediately after genotyping, MADM labeled embryonic brains were dissected and mounted in 4% low-melting agarose (Fisher BioReagents). A total of 300 μm coronal slices were prepared in oxygenated ice-cold artificial cerebrospinal fluid using a vibratome (Leica VT 1200S). Thereafter, slices were placed on Milicell culture inserts (Millipore) in 6-well glass-bottom dishes (MatTek) containing culture medium (1% 100X N2 supplement (Gibco), 1% Penicillin–Streptomycin (Gibco) in transparent F12/DMEM (Gibco)) and incubated (37°C, 5% CO_2_) for at least 45 min prior to imaging acquisition. To reduce the evaporation of media during imaging, a FoilCover lid (Pecon) was applied on top of the glass-bottom dishes during time-lapse imaging. A time-lapse of minimum 15 h with a frame rate of 15 ± min was recorded unidirectionally at seven Z-positions with 5 μm spacing using confocal microscopy [Zeiss LSM800, Plan-Apochromat 10×/0.45, WD = 2.1 mm objective, equipped with a heating chamber and stage-top incubator chamber and gas mixed (Ibidi) (37°C, 5% CO_2_)]. Excitation/emission wavelengths were 488/509 nm (EGFP) and 554/581 nm (tdTomato). Time-lapse images were collected on a PC running Zeiss ZEN Blue software. Time-lapse image series were Z-projected, time-stitched using ZEN blue software.

### Correction of non-linear local drift in time-lapse images

To correct any local tissue drift in the original 3D multi-channel movies, we developed the Python package undrift. First, dense optical flow from successive image pairs is estimated with the Farnebäck method [17] using the OpenCV library (version 3.3.1). For movies with more than one input channel, we used the averaged channel intensities before estimating the optical flow for each pixel. Input parameters for the Farnebäck method were set as follows: the number of image pyramid levels to 3, the averaging window size to 512 × 512 (px), the size of the pixel neighborhood used to find polynomial expansions to 5 px, the standard deviation of the Gaussian that is used to smooth derivatives to 0.4 px and the number of iterations per pyramid level to 3. Parameters were optimized to capture the movement of single cells and the locally coherent drift of tissue regions (if present). Then, the pairwise optical flow fields were smoothed (locally weighted averaged) with a spatio-temporal Gaussian (σ_t = 1 px and σ_xy = 25.6 px) using the scikit-image library (0.16.2). The strong spatial smoothing effectively removes movement on a small scale (single cells), whereas spatially coherent optical flow on a bigger scale (tissue drift) is maintained in the output. The smoothed pairwise optical flow fields are integrated over time to obtain an optical flow field relative to the reference frame (first time-point) using cubic spline interpolation and the Python SciPy library (version 1.4.1). New movies are rendered by artificially unwarping this integrated flow field on the original movie channels starting from the reference frame. For more information see http://github.com/hippenmeyerlab/undrift.

### Analysis of neuronal trajectories

Neurons were tracked semi-automatically with the ImageJ plugin TrackMate [78] using the LoG detector (estimated blob diameter: 10.0micron, threshold: 2.0, Median filter: enabled, sub-pixel localization: enabled) and the linear motion LAP tracker (initial search radius: 15, max search radius: 15, max frame gap: 2) for each channel. Tracks were manually curated to ensure correct tracking of neurons. Only red and green neuronal tracks were included in the analysis, all yellow neurons were excluded in the analysis. Tissue compartments (upper/lower zone) were drawn manually. All parameters were extracted in a .csv file for analysis. For each neuron, we first determined if it was located in the located in the upper/lower zone in the first frame or last frame. In Fig. 3E–F: each cell track was grouped into upper zone if in any frame the cell was positioned in the upper zone area. The cell track was grouped into lower zone if the cell was in no frame placed in the upper zone area. An arcsin conversion was performed of relative percentages for statistical calculations. Figures 3G–H: we extracted the x/y coordinates of cells based on their position (upper zone, lower zone). The resulting cells were re-grouped into cell tracks and for each track we calculated mean straight-line speed and directionality as follows.

Distance of one cell between two frames as:

$$ d\left({x}_i,{x}_j\right)=\sqrt{\sum_{d=1}^2{\left({x}_{i,d}-{x}_{j,d}\right)}^2} $$

Sum of all distances (total distance traveled) with N being the number of frames a cell was tracked in:

$$ {d}_{tot}={\sum}_{i=1}^{N-1}d\left({x}_i,{x}_{i+1}\right) $$

Net distance traveled:

$$ {d}_{net}=d\left({x}_{1,}{x}_N\right) $$

Net time traveled:

$$ {t}_{net}={t}_n-{t}_1 $$

Mean straight line speed:

$$ {d}_{net}/{t}_{net} $$

Directionality (meandering) index:

$$ {d}_{net}/{d}_{tot} $$

Note that the time between frames can differ for each cell (e.g. if a cell was not identified in one frame) and that the total time a cell was tracked can also differ between cells. Therefore, to assure the same frame rate for the analysis, we calculated the velocities in micrometer per minute. Note that a single track can be divided into sub-tracks if crossing the middle line. These tracks are ‘crossing’ and make up on average ~6% of all tracks. For Fig. 3E–H, we ranked each cell track from each video based on d_net_ and plotted the indicated values for the top 15 cells for each video. Statistical analyses were done with GraphPad Prism 8.0.1, applying a two-way ANOVA and a Tukey post hoc test.

### Formulation of computational migration model

The formulated 2D migration model is evaluated via a Python 3.6 script. The force generation mechanism is based on a random walk model with directional bias on a single cell of unit mass [10]. We include an alteration from [10] by generating a 2D force ${F}_{gen}$ in an intermediate step instead of directly calculating a displacement for each cell. The total force acting on a given cell at each time instance is given by:

$$ {F}_{tot}={F}_{gen}-{F}_{drag}+{F}_{spring} $$

For each timestep $dt$ the internal molecular machinery of a cell generated force ${F}_{gen}$. If this force is insufficiently high to overcome the subsequently resulting drag force ${F}_{drag}$ it is stored in form of a spring-like force ${F}_{spring}$ for the next timestep. Whereas the drag force is defined as:

$$ {F}_{drag}= c\eta v $$

Here, $\eta$ denotes the dynamic viscosity and $c$ is a parameter dependent on the cell shape, which we consider a spherical particle of unit radius. In our model both parameters are considered to be locally constant and can therefore be unified as a resistance parameter $R$. Similar models have been previously described for 3D cell migration in extra cellular matrices [85]. We include the cell intrinsic difference between control and KO-*Cdk5r1*-MADM in the form of a directionality parameter $\rho$ and a force scaling parameter $\alpha$ [10]. Here, a value of $\rho =0.5$ corresponds to a pure random walk model, which KO-*Cdk5r1*-MADM neurons tend towards. However, control neurons are closer to $\rho =1$, indicating a high directionality. The force scaling parameter $\alpha$ controls the magnitude of the generated force and henceforth velocity magnitudes. The non-cell-autonomous effect is included by introducing a linear coupling of directionality and force generation coefficient with the ratio $\beta$ of control cells $({N}_{Control})$ to mutant cells $({N}_{KO})$. Boundary values for $\alpha$ and $\rho$ at $\beta =0$ and $\beta =1$ correspond to values from experimental data for KO-*Cdk5r1*-MADM and control, respectively. For more details of our model and parameters see Table 1 below and Fig. S4.

**Table 1:** Modelling parameters

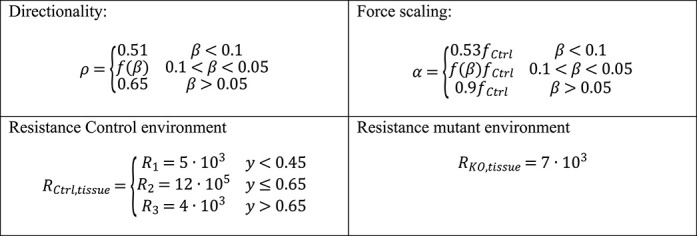

### Preparation of cell suspension and FACS

Preparation of cell suspension for cell sorting was prepared as previously described [46] for E13, E16 and P0 time points for RNA-seq and P0 for proteomics. From the overall MADM samples, GFP^+^ cells were collected for each genotype. For RNA-sequencing, 2000 cells per replicate (1 replicate=1 cerebral cortex) were sorted directly into a custom-made lysis buffer (30 nM TRIS pH 8, 10 nM EDTA pH 8, 1% SDS and 200 μg/μl Proteinase K). For proteomics, 10 000 cells per replicate (1 replicate = 1 cerebral cortex) were prepared as described above except no serum was added to the media and an extra wash step with DMD/F12 wash was carried out. Samples for proteomics were sorted directly into 50 μl lysis buffer (LYSE-NHS, Preomics iST-NHS kit).

### RNA extraction of MADM samples for RNA sequencing

Directly after cell sorting using FACS, samples were incubated for 30 min at 37°C. Total volume was filled to 250 μl using RNase-free H2O (Thermo Fisher Scientific) followed by the addition of 750 μl Trizol LS (Thermo Fisher Scientific). Samples were mixed by inversion (five times). After a 5 min incubation step at RT, the entire solution was transferred into a MaXtract tube (QIAGEN). A total of 200 μl chloroform (Sigma-Aldrich) was added, followed by three times 5 s vortexing and 2 min incubation at RT. Samples were centrifuged for 2 min at 12 000 rpm at 18°C. The supernatant was transferred to a new tube and isopropanol (Sigma-Aldrich) was added in a 1:1 ratio. For better visibility of the RNA pellet 1 μl GlycoBlue (Thermo Fisher Scientific) was added and the entire solution was mixed by vortexing (3× 5 s). Samples were left for precipitation o/n at −20°C. After precipitation samples were centrifuged for 20 min with 14 000 rpm at 4°C. The supernatant was removed and the RNA pellet was washed with 70% ethanol, followed by a 5 min centrifugation step (14 000 rpm at 4°C). The RNA pellet was resuspended in 12.5 μl RNase-free H_2_O. RNA quality was analysed using RNA 6000 Pico kit (Agilent) following the manufacturer’s instructions. The RNA samples were stored at −80°C until further use. RNA sequencing was performed by VBCF GmbH on Illumina platforms.

### Statistical analysis of RNA-Seq

Read processing, alignment and annotations are described elsewhere [45]. STAR alignment parameters: clip5pNbases 3, outFilterMultimapNmax 1, outSAMtype BAM SortedByCoordinate and quantMode GeneCounts. Downstream analyses were performed in R (v3.6.1). Read coverages of the deleted *Cdk5r1* region (chr11:80477417–80478722, mm10) and the deleted *Dab1* region (chr4:104605298–104605437, mm10) were calculated using bedtools intersect with the split option on the aligned bam file produced by STAR. These read counts were added to the count tables produced by STAR with the gene name *Cdk5r1*_del and *Dab1*_del, respectively.

For Fig. 5A–E, we analysed 81 samples and removed 9 samples with a low percentage of uniquely aligned reads (<50%) or due to their position on the PCA plot. Statistics on differential expression between all pairs of genotypes were calculated with DESeq2 (v1.26.0) using contrasts for each developmental time point separately. To reduce noise, only genes with an average read coverage of >10 were used in the analyses. We used an adjusted *P*-value (padj) cutoff of 0.05 for DEGs for all analyses in Fig. 5A–E. Up- and down-regulated genes were determined by a log_2_ fold-change of >0 or < 0, respectively. For Gene Ontology term enrichment, we used the enrichGO function from the clusterProfiler package (v3.14.0) with parameters: universe = [all informative genes in the respective comparison], ont = ‘ALL’, pool = T, readable = T, OrgDb = org.Mm.eg.db (v3.10.0), minGSSize = 20, maxGSSize = 500, *P*-value cutoff = 0.1, qvalueCutoff = 0.2. For the GO term plot in Fig. 5E, we focused on P0 timepoint and first removed GO terms that are not related to neuronal development by removing GO terms with blood, vascu or angio in their description. Then we calculated the negative log10 of the uncorrected *P*-value for the remaining GO terms (score). Finally, we ranked GO terms by the score and plotted the score of the top 10 GO terms focusing on the ontology BP.

For Fig. 7, we analysed 38 samples consisting of 22 samples already used for Fig. 5A–E (control, *KO-Cdk5r1-MADM*, P0 time point) and 16 new samples (control, *Dab1*-KO). We removed three samples due to their position on a PCA plot. Statistics on differential expression were calculated as for Fig. 5A–E using genes with an average read coverage over all samples >20. For all analyses in Fig. 7, we used an adjusted *P*-value (padj) cutoff of 0.05 and an absolute log_2_ fold-change >0.35 to define DEGs. Figure 7E: We calculated the % common up- and down-regulated genes relative to all DEG in KO-*Dab1*/control and *KO-Cdk5r1-MADM*/control, respectively, and plotted the mean of these two values. Figure 7F: significance of the overlap between DEG groups was calculated using newGOM from package GeneOverlap (v1.22.0) using the number of informative genes in this comparison as genome.size. We used different gene groups for further analysis: Common_up defines genes that are common up-regulated DEGs (intersection Fig. 7D left), Common_down defines genes that are common down-regulated DEGs (intersection Fig. 7D right), Dab1_spec defines genes that are DEG in *Dab1*-KO/control but not in *KO-Cdk5r1-MADM*/control (*Dab1* up, *Dab1* down in Fig. 7D) and *Cdk5r1*_spec defines genes that are DEG in *KO-Cdk5r1-MADM*/control but do not overlap (*Cdk5r1* up, *Cdk5r1* down Fig. 7D). GO term enrichment for the respective gene group was performed using enrichGO with parameters: enrichGO(OrgDb = org.Mm.eg.db, readable = T, pool = T, maxGSSize = 900, minGSSize = 100, *P*-value cutoff = 0.05, qvalueCutoff = 0.1, separately for different GO ontologies. For Fig. 7G, we calculated a score as in Fig. 7E and plotted the top 10 GO terms from cellular components (CC) ontology without prior filtering. For Fig. S8 we used the used top 50 GO terms (ranked by adjusted *P*-value) from *Dab1*_spec and *Cdk5r1*_spec analysis and calculated all pairwise semantic similarity goSim from GOSemSim (v2.12.1) package with parameters: measure = ‘Jiang’. We plotted the resulting similarity matrix using the pheatmap package.

### Sample processing for proteomics

Samples were divided into two batches, each containing three control samples and five *Cdk5r1*-MADM or KO-*Cdk5r1*-MADM samples. Batch 1, processed on day 1, contained green cells from two individual litters and corresponded to control versus KO-*Cdk5r1*-MADM comparison; batch 2, processed on day 2, contains green from three individual litters and was used for the control versus *Cdk5r1*-MADM comparison. Each litter contained both control and either *Cdk5r1*-MADM or KO-*Cdk5r1*-MADM. Protein extraction, tryptic digestion and peptides cleanup were performed using a TMT-labeling compatible variant of the in-Stage Tips method (iST-NHS-12x kit, Preomics). Briefly, immediately after cell-sorting, collected cells were supplemented with 50 μl LYSE-NHS buffer, boiled for 10 min, then processed according to the manufacturer’s protocol with the following minor modifications: sonication was skipped (the number of cells was low enough that DNA would not be an issue, and this would reduce the chance of proteins loss or samples contamination due to having to use a probe sonicator); digestion was performed overnight. Prior to TMT labeling, small aliquots of each of the 16 samples were taken and mixed to generate a mixed reference sample. Individual samples were then labeled with TMT-10plex (lot # UL291039, ThermoFisher Scientific), splitting the contents of each TMT vial to label one sample of each batch. Individual samples were combined into two TMT-labeled samples, each containing the eight samples from one batch plus a mixed reference sample. Combined samples were then loaded onto the iST-NHS kit’s cartridges in several steps, washed as per the manufacturer’s protocol, eluted and dried in a speedvac. Since phospho-peptides were of interest, although the amount of material as determined by a Pierce Quantitative Colorimetric Peptide Assay (Thermo Scientific) was low (~100 μg/sample), the samples were subjected to phospho-peptides enrichment (MagReSyn Ti-IMAC beads, ReSyn Biosciences) according to manufacturer’s protocol but scaling down the amount of beads, then the flow-throughs were fractionated into eight fractions using the Pierce High pH Reversed-Phase Peptide Fractionation Kit (ThermoFisher Scientific).

### LC–MS/MS analysis

Samples were dried, redissolved in 0.1% TFA and analysed by LC–MS/MS on an Ultimate HPLC (ThermoFisher Scientific) coupled to a Q-Exactive HF (ThermoFisher Scientific). Each sample was concentrated over an Acclaim PepMap C18 pre-column (5 μm particle size, 0.3 mm ID × 5 mm length, ThermoFisher Scientific) then bound to a 50 cm EasySpray C18 analytical column (2 μm particle size, 75 μm ID × 500 mm length, ThermoFisher Scientific) and eluted over the following 90 min gradient: solvent A, water +0.1% formic acid; solvent B, 80% acetonitrile in water +0.08% formic acid; constant 300 nl/min flow; B percentage: start, 2%; 70 min, 31%; 90 min, 44%. Mass spectra were acquired in positive mode with a data-dependent acquisition method: FWHM 20s, lock mass 445.12003 m/z; MS1: profile mode, 120 000 resolution, AGC target 3e6, 50 ms maximum IT, 380 to 1500 m/z; MS2: top 20, centroid mode, 1 microscan, 60 000 resolution, AGC target 1e5, 100 ms maximum IT, 0.7 m/z isolation window (no offset), 100 m/z fixed first mass, NCE 32, excluding charges 1 and 8 or higher, 60s dynamic exclusion.

### Statistical analysis of proteomics

Raw files were searched in MaxQuant 1.6.14.0 against the *Mus musculus* reference proteome from UniProtKB. Fixed cysteine modification was set to H11OC6N. Variable modifications were Oxidation (M), Acetyl (Protein N-term), Deamidation (NQ), Gln- > pyro-Glu and Phospho (STY). Match between runs and second peptides search were active. All FDRs were set to 1%. MaxQuant results were further processed in R using in-house scripts, starting from evidence (PSM) tables. Briefly, potential contaminants, reverse database hits or evidences with null intensity values were excluded. Evidence reporter intensities were scaled to integrated feature intensity, normalized using the Levenberg–Marquardt procedure row-wise, then assembled into peptidoforms (post-translationally modified peptides), summing up intensities per sample. Peptidoform reporter intensities were corrected for TMT lot label impurity values, median normalized, log-transformed, subjected to variance stabilizing normalization, re-normalized using the Levenberg–Marquardt procedure row-wise, then corrected for TMT batch effect using the Internal Reference Scaling method. Ratios to the average of all either control or *Cdk5r1*-MADM samples (two parallel analyses) were calculated, then protein groups were inferred from peptidoforms. Because the focus was on discovering and quantifying as many protein groups as possible from limiting sample amounts, all groups including those with just one peptide were retained. Protein groups were quantified by averaging the intensity profile of matching peptidoforms (excluding phospho-peptides and counterparts), weighted by the inverse of individual posterior error probabilities, then these values were averaged per sample and *P*-values calculated using a moderated t-test and an F-test (limma package). Significance thresholds were calculated using the Benjamini Hochberg procedure for 10%, 20% and 30% FDR. In addition, protein groups with an absolute log2 ratio smaller than 95% of individual to average reference log2 ratios were excluded. Results were saved as an EXCEL file and used for downstream analyses. Figures 5G–H: we plotted the Moderated.t-test:.-log10(*P*-value) against the Ratio:.log2.-.Mean for the respective comparison. All genes that were marked ‘up, FDR = 10%’ and ‘down, FDR = 10%’ were labeled in the volcano plot. For Fig. 5I: we used all gene names linked to peptide groups for subsequent analyses. We defined significant DEGs as genes marked ‘up, FDR = 10%’, ‘down, FDR = 10%’ and calculated GO term enrichments using clusterProfiler (v3.14.3) with parameters: universe = [all genes in ‘Genes’], OrgDb = org.Mm.eg.db (v3.10.0), ont = ‘CC’, *P*-value cutoff = 0.1, minGSSize = 50, maxGSSize = 2000, pool = F, readable = T. We plotted selected terms of the top 15 GO terms, ranked by *P*-value. Figures 5J and K: comparison to RNA-Seq: we defined RNA-Seq DEGs by using statistics calculated in Fig. 5B and extracting genes with an adjusted *P*-value of <0.1 and a log2 fold-change >0 (RNA Up) or <0 (RNA Down). DEGs based on proteomics were defined as having a ‘+’ in the ‘Significant:.FDR = 10%.-.full-KO’ column and ‘Ratio:.log2.-.Mean.-.full-KO’ <0 (Protein Down) or >0 (Protein Up). Note that we only used genes that were informative on both RNA-Seq and Proteomics for this analysis. Figure 5L: for GO term analysis, we used gene sets commonly up- and down-regulated in both RNA-Seq and proteomics (intersection Fig. 5K). GO term enrichment was calculated using enrichGO with parameters: universe = [all genes informative in RNA-Seq and proteomics], OrgDb = org.Mm.eg.db (v3.10.0), ont = ‘CC’, *P*-value cutoff = 0.9, minGSSize = 10, maxGSSize = 2000, pool = F, readable = T. We calculated a score as described before with a negative prefix for down regulated genes and plotted the top 10 GO terms, ranked by *P*-value.

### Transcriptomics raw data

The raw sequencing data used in this publication were deposited in NCBI’s Gene Expression Omnibus [15] and be accessible through GEO Series accession number GSE200604 (https://www.ncbi.nlm.nih.gov/geo/query/acc.cgi?acc=GSE200604).

### Proteomics raw data

The mass spectrometry proteomics data have been deposited to the ProteomeXchange Consortium via the PRIDE [61] partner repository with the dataset identifier PXD023222.

### Code for analysis of relative distribution of MADM-Labeled neurons

The Python (version > = 3.6) package cell2layer (version 0.2) for analysing the relative and absolute distances of each manually marked neuron to its layer boundaries along with documentation is available at http://github.com/hippenmeyerlab/cell2layer as open-source software under the GNU General Public License v3.0.

### Code for correction of non-linear local drift in time-lapse images

The Python (version > = 3.6) package undrift (version 0.2) for correcting non-linear, local tissue drift along with documentation is available at http://github.com/hippenmeyerlab/undrift as open-source software under the GNU General Public License v3.0.

## Supplementary Material

suppl_data_kvac009

## Data Availability

All data have been presented in Figures and Supplemental Figures. Original images will be made available upon request. Raw sequencing/proteomics data, sample lists and code will be accessible as detailed.

## References

[1] Amberg N, Hippenmeyer S. Genetic mosaic dissection of candidate genes in mice using mosaic analysis with double markers. STAR Protoc. 2021;2: 100939 10.1016/j.xpro.2021.100939 PMC8603308 34825212

[2] Angevine JB, Sidman RL. Autoradiographic study of cell migration during histogenesis of cerebral cortex in the mouse. Nature. 1961;192:766–8 10.1038/192766b0 17533671

[3] Arnaud L, Ballif BA, Cooper JA. Regulation of protein tyrosine kinase signaling by substrate degradation during brain development. Mol. Cell. Biol. 2003;23:9293–302 14645539 10.1128/MCB.23.24.9293-9302.2003 PMC309695

[4] Ayala R, Shu T, Tsai LH. Trekking across the brain: the journey of neuronal migration. Cell. 2007;128:29–43 17218253 10.1016/j.cell.2006.12.021

[5] Baek ST, Copeland B, Yun E-J et al. An AKT3-FOXG1-reelin network underlies defective migration in human focal malformations of cortical development. Nat Med. 2015;21:1445–54 26523971 10.1038/nm.3982 PMC4955611

[6] van den Berghe V, Stappers E, Seuntjens E. How cell-autonomous is neuronal migration in the forebrain? Molecular cross-talk at the cell membrane. Neurosci. 2014;20:571–5 10.1177/1073858414539396 24972605

[7] Bock HH, May P. Canonical and non-canonical Reelin signaling. Front Cell Neurosci. 2016;10:1–20 27445693 10.3389/fncel.2016.00166 PMC4928174

[8] Bradshaw NJ, Hayashi MAF. NDE1 and NDEL1 from genes to (mal)functions: parallel but distinct roles impacting on neurodevelopmental disorders and psychiatric illness. Cell Mol Life Sci. 2017;74:1191–210 27742926 10.1007/s00018-016-2395-7 PMC11107680

[9] Buchsbaum IY, Cappello S. Neuronal migration in the CNS during development and disease: insights from in vivo and in vitro models. Development. 2019;146:10.1242/dev.163766 30626593

[10] Caffrey JR, Hughes BD, Britto JM, Landman KA. An in silico agent-based model demonstrates Reelin function in directing lamination of neurons during cortical development. Plos One. 2014;9:1–11 10.1371/journal.pone.0110415 PMC4204858 25334023

[11] Chae T, Kwon YT, Bronson R et al. Mice lacking p35, a neuronal specific activator of Cdk5, display cortical lamination defects, seizures, and adult lethality. Neuron. 1997;18:29–42 9010203 10.1016/s0896-6273(01)80044-1

[12] Contreras X, Amberg N, Davaatseren A et al. A genome-wide library of MADM mice for single-cell genetic mosaic analysis. Cell Rep. 2021;35:109274 34161767 10.1016/j.celrep.2021.109274 PMC8317686

[13] Delalle I, Bhide PG, Caviness VS, Tsai LH. Temporal and spatial patterns of expression of p35, a regulatory subunit of cyclin-dependent kinase 5, in the nervous system of the mouse. J Neurocytol. 1997;26:283–96 9192293 10.1023/a:1018500617374

[14] Dimidschstein J, Passante L, Dufour A et al. Ephrin-B1 controls the columnar distribution of cortical pyramidal neurons by restricting their tangential migration. Neuron. 2013;79:1123–35 24050402 10.1016/j.neuron.2013.07.015

[15] Edgar R. Gene Expression Omnibus: NCBI gene expression and hybridization array data repository. Nucleic Acids Res. 2002;30:207–10 11752295 10.1093/nar/30.1.207 PMC99122

[16] Evsyukova I, Plestant C, Anton ES. Integrative mechanisms of oriented neuronal migration in the developing brain. Annu Rev Cell Dev Biol. 2013;29:299–353 23937349 10.1146/annurev-cellbio-101512-122400 PMC3930923

[17] Farnebäck G. Two-frame motion estimation based on polynomial expansion. Lect Notes Comput Sci (Including Subser Lect Notes Artif Intell Lect Notes Bioinformatics). 2003;2749:363–70

[18] Franco SJ, Martinez-Garay I, Gil-Sanz C et al. Reelin regulates cadherin function via Dab1/Rap1 to control neuronal migration and lamination in the neocortex. Neuron. 2011;69:482–97 21315259 10.1016/j.neuron.2011.01.003 PMC3056352

[19] Gorelik A, Sapir T, Woodruff TM, Reiner O. Serping1/C1 inhibitor affects cortical development in a cell autonomous and non-cell autonomous manner. Front Cell Neurosci. 2017;11:1–14 28670268 10.3389/fncel.2017.00169 PMC5472692

[20] Gorski JA, Talley T, Qiu M et al. Cortical excitatory neurons and glia, but not GABAergic neurons, are produced in the Emx1-expressing lineage. J Neurosci. 2002;22:6309–14 12151506 10.1523/JNEUROSCI.22-15-06309.2002 PMC6758181

[21] Greenman R, Gorelik A, Sapir T et al. Non-cell autonomous and non-catalytic activities of ATX in the developing brain. Front Neurosci. 2015;9:1–17 25788872 10.3389/fnins.2015.00053 PMC4349085

[22] Guerrini R, Parrini E. Neuronal migration disorders. Neurobiol Dis. 2010;38:154–66 19245832 10.1016/j.nbd.2009.02.008

[23] Gupta A, Sanada K, Miyamoto DT et al. Layering defect in p35 deficiency is linked to improper neuronal-glial interaction in radial migration. Nat Neurosci. 2003;6:1284–91 14608361 10.1038/nn1151

[24] Hammond V, Howell B, Godinho L, Tan SS. Disabled-1 functions cell autonomously during radial migration and cortical layering of pyramidal neurons. J Neurosci. 2001;21:8798–808 11698592 10.1523/JNEUROSCI.21-22-08798.2001 PMC6762297

[25] Hammond V, Tsai LH, Tan SS. Control of cortical neuron migration and layering: cell and non cell-autonomous effects of p35. J Neurosci. 2004;24:576–87 14724258 10.1523/JNEUROSCI.4529-03.2004 PMC6729984

[26] Hanganu-Opatz IL, Butt SJB, Hippenmeyer S et al. The logic of developing neocortical circuits in health and disease. J Neurosci. 2021;41:813–22 33431633 10.1523/JNEUROSCI.1655-20.2020 PMC7880298

[27] Hansen AH, Duellberg C, Mieck C et al. Cell polarity in cerebral cortex development—cellular architecture shaped by biochemical networks. Front Cell Neurosci. 2017;11:176 28701923 10.3389/fncel.2017.00176 PMC5487411

[28] Hansen AH, Hippenmeyer S. Non-cell-autonomous mechanisms in radial projection neuron migration in the developing cerebral cortex. Front Cell Dev Biol. 2020;8: 574382 10.3389/fcell.2020.574382 PMC7545535 33102480

[29] Hatanaka Y, Hisanaga S-I, Heizmann CW, Murakami F. Distinct migratory behavior of early- and late-born neurons derived from the cortical ventricular zone. J Comp Neurol. 2004;479:1–14 15389616 10.1002/cne.20256

[30] Heng JIT, Chariot A, Nguyen L. Molecular layers underlying cytoskeletal remodelling during cortical development. Trends Neurosci. 2010;33:38–47 19837469 10.1016/j.tins.2009.09.003

[31] Hippenmeyer S. Molecular pathways controlling the sequential steps of cortical projection neuron migration. Cell Mol Control Neuron Migrat. 2014;800:1–24 10.1007/978-94-007-7687-6_1 24243097

[32] Hippenmeyer S, Youn YH, Moon HM et al. Genetic mosaic dissection of Lis1 and Ndel1 in neuronal migration. Neuron. 2010;68:695–709 21092859 10.1016/j.neuron.2010.09.027 PMC3044607

[33] Hong SE, Shugart YY, Huang DT et al. Autosomal recessive lissencephaly with cerebellar hypoplasia is associated with human RELN mutations. Nat Genet. 2000;26:93–6 10973257 10.1038/79246

[34] Howell BW, Hawkes R, Soriano P, Cooper JA. Neuronal position in the developing brain is regulated by mouse disabled-1. Nature. 1997;389:733–7 9338785 10.1038/39607

[35] Iwashita M, Kataoka N, Toida K, Kosodo Y. Systematic profiling of spatiotemporal tissue and cellular stiffness in the developing brain. Development. 2014;141:3793–8 25249464 10.1242/dev.109637

[36] Jheng G-W, Hur SS, Chang C-M et al. Lis1 dysfunction leads to traction force reduction and cytoskeletal disorganization during cell migration. Biochem Biophys Res Commun. 2018;497:869–75 29470990 10.1016/j.bbrc.2018.02.151

[37] Jossin Y. Molecular mechanisms of cell polarity in a range of model systems and in migrating neurons. Mol Cell Neurosci. 2020;106: 103503 10.1016/j.mcn.2020.103503 32485296

[38] Juric-Sekhar G, Hevner RF. Malformations of cerebral cortex development: molecules and mechanisms. Annu Rev Pathol. 2019;14:293–318 30677308 10.1146/annurev-pathmechdis-012418-012927 PMC6938687

[39] Keshvara L, Magdaleno S, Benhayon D et al. Cyclin-dependent kinase 5 phosphorylates disabled 1 independently of Reelin signaling. J Neurosci. 2002;22:4869–77 12077184 10.1523/JNEUROSCI.22-12-04869.2002 PMC6757745

[40] Ko J, Humbert S, Bronson RT et al. p35 and p39 are essential for cyclin-dependent kinase 5 function during neurodevelopment. J Neurosci. 2001;21:6758–71 11517264 10.1523/JNEUROSCI.21-17-06758.2001 PMC6763073

[41] Kriegstein AR, Noctor SC. Patterns of neuronal migration in the embryonic cortex. Trends Neurosci. 2004;27:392–9 15219738 10.1016/j.tins.2004.05.001

[42] Kwan KY, Sestan N, Anton ES. Transcriptional co-regulation of neuronal migration and laminar identity in the neocortex. Development. 2012;139:1535–46 22492350 10.1242/dev.069963 PMC3317962

[43] Kwon YT, Gupta A, Zhou Y et al. Regulation of N-cadherin-mediated adhesion by the p35-Cdk5 kinase. Curr Biol. 2000;10:363–72 10753743 10.1016/s0960-9822(00)00411-5

[44] Kwon YT, Tsai LH. A novel disruption of cortical development in p35(−/−) mice distinct from reeler. J Comp Neurol. 1998;395:510–22 9619503 10.1002/(sici)1096-9861(19980615)395:4<510::aid-cne7>3.0.co;2-4

[45] Laukoter S, Amberg N, Pauler FM, Hippenmeyer S. Generation and isolation of single cells from mouse brain with mosaic analysis with double markers-induced uniparental chromosome disomy. STAR Protoc. 2020a;1: 100215 10.1016/j.xpro.2020.100215 PMC7757670 33377108

[46] Laukoter S, Beattie R, Pauler FM et al. Imprinted Cdkn1c genomic locus cell-autonomously promotes cell survival in cerebral cortex development. Nat Commun. 2020b;11:1–14 31924768 10.1038/s41467-019-14077-2 PMC6954230

[47] Lee JH, Huynh M, Silhavy JL et al. De novo somatic mutations in components of the PI3K-AKT3-mTOR pathway cause hemimegalencephaly. Nat Genet. 2012;44:941–5 22729223 10.1038/ng.2329 PMC4417942

[48] Lodato S, Arlotta P. Generating neuronal diversity in the mammalian cerebral cortex. Annu Rev Cell Dev Biol. 2015;31:699–720 26359774 10.1146/annurev-cellbio-100814-125353 PMC4778709

[49] Magen D, Ofir A, Berger L et al. Autosomal recessive lissencephaly with cerebellar hypoplasia is associated with a loss-of-function mutation in CDK5. Hum Genet. 2015;134:305–14 25560765 10.1007/s00439-014-1522-5

[50] Mähler (Convenor M, Berard M, Feinstein R et al. FELASA recommendations for the health monitoring of mouse, rat, hamster, guinea pig and rabbit colonies in breeding and experimental units. Lab Anim. 2014;48:178–92 24496575 10.1177/0023677213516312

[51] Marín O, Valiente M, Ge X, Tsai LH. Guiding neuronal cell migrations. Cold Spring Harb Perspect Biol. 2010;2:1–21 10.1101/cshperspect.a001834 PMC2828271 20182622

[52] McConnell SK. Constructing the cerebral cortex: neurogenesis and fate determination. Neuron. 1995;15:761–8 7576626 10.1016/0896-6273(95)90168-x

[53] Morimura T, Ogawa M et al. Relative importance of the tyrosine phosphorylation sites of Disabled-1 to the transmission of Reelin signaling. Brain Res. 2009;1304:26–37 19796633 10.1016/j.brainres.2009.09.087

[54] Nadarajah B, Brunstrom JE, Grutzendler J et al. Two modes of radial migration in early development of the cerebral cortex. Nat Neurosci. 2001;4:143–50 11175874 10.1038/83967

[55] Nakagawa N, Plestant C, Yabuno-Nakagawa K et al. Memo1-mediated tiling of radial glial cells facilitates cerebral cortical development. Neuron. 2019;103:836–852.e5 31277925 10.1016/j.neuron.2019.05.049 PMC6728225

[56] Niethammer M, Smith DS, Ayala R et al. NUDEL is a novel Cdk5 substrate that associates with LIS1 and cytoplasmic dynein. Neuron. 2000;28:697–711 11163260 10.1016/s0896-6273(00)00147-1

[57] Noctor SC, Flint AC, Weissman TA et al. Neurons derived from radial glial cells establish radial units in neocortex. Nature. 2001;409:714–20 11217860 10.1038/35055553

[58] Noctor SC, Martínez-Cerdeño V, Ivic L, Kriegstein AR. Cortical neurons arise in symmetric and asymmetric division zones and migrate through specific phases. Nat Neurosci. 2004;7:136–44 14703572 10.1038/nn1172

[59] Ohshima T. Neuronal migration and protein kinases. Front Neurosci. 2015;8:458 25628530 10.3389/fnins.2014.00458 PMC4292441

[60] Patrick GN, Zhou P, Kwon YT et al. p35, the neuronal-specific activator of cyclin-dependent kinase 5 (Cdk5) is degraded by the ubiquitin-proteasome pathway. J Biol Chem. 1998;273:24057–64 9727024 10.1074/jbc.273.37.24057

[61] Perez-Riverol Y, Csordas A, Bai J et al. The PRIDE database and related tools and resources in 2019: improving support for quantification data. Nucleic Acids Res. 2019;47:D442–50 30395289 10.1093/nar/gky1106 PMC6323896

[62] Poduri A, Evrony GD, Cai X et al. Somatic activation of AKT3 causes hemispheric developmental brain malformations. Neuron. 2012;74:41–8 22500628 10.1016/j.neuron.2012.03.010 PMC3460551

[63] Pramparo T, Libiger O, Jain S et al. Global developmental gene expression and pathway analysis of normal brain development and mouse models of human neuronal migration defects. PLoS Genet. 2011;7: e1001331 10.1371/journal.pgen.1001331 PMC3053345 21423666

[64] Reiner O, Parichha A, Sapir T. Modeling human neuronal migration deficits in 3D. Curr Opin Neurobiol. 2021;66:30–6 33069990 10.1016/j.conb.2020.09.005

[65] Reiner O, Sapir T. LIS1 functions in normal development and disease. Curr Opin Neurobiol. 2013;23:951–6 23973156 10.1016/j.conb.2013.08.001

[66] Rivière JB, Mirzaa GM, O’Roak BJ et al. De novo germline and postzygotic mutations in AKT3, PIK3R2 and PIK3CA cause a spectrum of related megalencephaly syndromes. Nat Genet. 2012;44:934–40 22729224 10.1038/ng.2331 PMC3408813

[67] Samuels BA, Hsueh Y-P, Shu T et al. Cdk5 promotes synaptogenesis by regulating the subcellular distribution of the MAGUK family member CASK. Neuron. 2007;56:823–37 18054859 10.1016/j.neuron.2007.09.035 PMC2151975

[68] Sanada K, Gupta A, Tsai LH. Disabled-1-regulated adhesion of migrating neurons to radial glial fiber contributes to neuronal positioning during early corticogenesis. Neuron. 2004;42:197–211 15091337 10.1016/s0896-6273(04)00222-3

[69] Sasaki S, Shionoya A, Ishida M et al. A LIS1/NUDEL/cytoplasmic dynein heavy chain complex in the developing and adult nervous system. Neuron. 2000;28:681–96 11163259 10.1016/s0896-6273(00)00146-x

[70] Schindelin J, Arganda-Carreras I, Frise E et al. Fiji: an open-source platform for biological-image analysis. Nat Methods. 2012;9:676–82 22743772 10.1038/nmeth.2019 PMC3855844

[71] Sekine K, Honda T, Kawauchi T et al. The outermost region of the developing cortical plate is crucial for both the switch of the radial migration mode and the Dab1-dependent “inside-out” lamination in the neocortex. Rapid Commun. 2011;31:9426–39 10.1523/JNEUROSCI.0650-11.2011 PMC6623472 21697392

[72] Sekine K, Kawauchi T, Kubo K et al. Reelin controls neuronal positioning by promoting cell-matrix adhesion via inside-out activation of integrin α5β1. Neuron. 2012;76:353–69 23083738 10.1016/j.neuron.2012.07.020 PMC3479437

[72] Shellard A, Mayor R. Rules of collective migration: from the wildebeest to the neural crest. Decennial index. 2020;375: 20190387 10.1098/rstb.2019.0387 PMC7423382 32713298

[74] Simo S, Jossin Y, Cooper JA. Cullin 5 regulates cortical layering by modulating the speed and duration of Dab1-dependent neuronal migration. J Neurosci. 2010;30:5668–76 20410119 10.1523/JNEUROSCI.0035-10.2010 PMC2866641

[75] Su SC, Tsai L-H. Cyclin-dependent kinases in brain development and disease. Annu Rev Cell Dev Biol. 2011;27:465–91 21740229 10.1146/annurev-cellbio-092910-154023

[76] Tabata H, Nakajima K. Multipolar migration: the third mode of radial neuronal migration in the developing cerebral cortex. J Neurosci. 2003;23:9996–10001 14602813 10.1523/JNEUROSCI.23-31-09996.2003 PMC6740853

[77] Takahashi T, Nowakowski R, Caviness V. The cell cycle of the pseudostratified ventricular epithelium of the embryonic murine cerebral wall. J Neurosci. 1995;15:6046–57 7666188 10.1523/JNEUROSCI.15-09-06046.1995 PMC6577667

[78] Tinevez J-Y, Perry N, Schindelin J et al. TrackMate: an open and extensible platform for single-particle tracking. Methods. 2017;115:80–90 27713081 10.1016/j.ymeth.2016.09.016

[79] Tsai J, Vallee RB Stem Cell Migration. Totowa, NJ: Humana Press, 2011

[80] Valiente M, Marín O. Neuronal migration mechanisms in development and disease. Curr Opin Neurobiol. 2010;20:68–78 20053546 10.1016/j.conb.2009.12.003

[81] Vallee RB, Seale GE, Tsai J-W. Emerging roles for myosin II and cytoplasmic dynein in migrating neurons and growth cones. Trends Cell Biol. 2009;19:347–55 19524440 10.1016/j.tcb.2009.03.009 PMC2844727

[82] Yang H, Jensen P, Goldowitz D. The community effect and Purkinje cell migration in the cerebellar cortex: analysis of scrambler chimeric mice. J Neurosci. 2002;22:464–70 11784791 10.1523/JNEUROSCI.22-02-00464.2002 PMC6758652

[83] Yoshinaga S, Honda T, Kubo K, Nakajima K. Dab1-deficient deep layer neurons prevent Dab1-deficient superficial layer neurons from entering the cortical plate. Neurosci Res. 2022; 10.1016/j.neures.2022.03.011 35364133

[84] Youn YH, Pramparo T, Hirotsune S, Wynshaw-Boris A. Distinct dose-dependent cortical neuronal migration and neurite extension defects in Lis1 and Ndel1 mutant mice. J Neurosci. 2009;29:15520–30 20007476 10.1523/JNEUROSCI.4630-09.2009 PMC2824645

[85] Zaman MH, Kamm RD, Matsudaira P, Lauffenburger DA. Computational model for cell migration in three-dimensional matrices. Biophys J. 2005;89:1389–97 15908579 10.1529/biophysj.105.060723 PMC1366623

[86] Zong H, Espinosa JS, Su HH et al. Mosaic analysis with double markers in mice. Cell. 2005;121:479–92 15882628 10.1016/j.cell.2005.02.012

